# Tight junction protein cingulin variant is associated with cancer susceptibility by overexpressed IQGAP1 and Rac1-dependent epithelial-mesenchymal transition

**DOI:** 10.1186/s13046-024-02987-z

**Published:** 2024-03-01

**Authors:** Yi-Ting Huang, Ya-Ting Hsu, Pei-Ying Wu, Yu-Min Yeh, Peng-Chan Lin, Keng-Fu Hsu, Meng-Ru Shen

**Affiliations:** 1 Department of Pharmacology, College of Medicine, National Cheng Kung University, Tainan, Taiwan; 2 Institute of Basic Medical Sciences, College of Medicine, National Cheng Kung University, Tainan, Taiwan; 3 Institute of Clinical Medicine, College of Medicine, National Cheng Kung University, Tainan, Taiwan; 4 Division of Hematology, Department of Internal Medicine, National Cheng Kung University Hospital, College of Medicine, National Cheng Kung University, Tainan, Taiwan; 5 Department of Obstetrics and Gynecology, National Cheng Kung University Hospital, College of Medicine, National Cheng Kung University, Tainan, Taiwan; 6 Department of Oncology, National Cheng Kung University Hospital, College of Medicine, National Cheng Kung University, Tainan, Taiwan

**Keywords:** Cingulin, Rac1, IQGAP1, Epithelial-mesenchymal transition, Cancer-predisposing variant

## Abstract

**Background:**

Cingulin (CGN) is a pivotal cytoskeletal adaptor protein located at tight junctions. This study investigates the link between *CGN* mutation and increased cancer susceptibility through genetic and mechanistic analyses and proposes a potential targeted therapeutic approach.

**Methods:**

In a high-cancer-density family without known pathogenic variants, we performed tumor-targeted and germline whole-genome sequencing to identify novel cancer-associated variants. Subsequently, these variants were validated in a 222 cancer patient cohort, and *CGN* c.3560C > T was identified as a potential cancer-risk allele. Both wild-type (WT) (c.3560C > C) and variant (c.3560C > T) were transfected into cancer cell lines and incorporated into orthotopic xenograft mice model for evaluating their effects on cancer progression. Western blot, immunofluorescence analysis, migration and invasion assays, two-dimensional gel electrophoresis with mass spectrometry, immunoprecipitation assays, and siRNA applications were used to explore the biological consequence of *CGN* c.3560C > T.

**Results:**

In cancer cell lines and orthotopic animal models, *CGN* c.3560C > T enhanced tumor progression with reduced sensitivity to oxaliplatin compared to the *CGN* WT. The variant induced downregulation of epithelial marker, upregulation of mesenchymal marker and transcription factor, which converged to initiate epithelial-mesenchymal transition (EMT). Proteomic analysis was conducted to investigate the elements driving EMT in *CGN* c.3560C > T. This exploration unveiled overexpression of IQGAP1 induced by the variant, contrasting the levels observed in *CGN* WT. Immunoprecipitation assay confirmed a direct interaction between CGN and IQGAP1. IQGAP1 functions as a regulator of multiple GTPases, particularly the Rho family. This overexpressed IQGAP1 was consistently associated with the activation of Rac1, as evidenced by the analysis of the cancer cell line and clinical sample harboring *CGN* c.3560C > T. Notably, activated Rac1 was suppressed following the downregulation of IQGAP1 by siRNA. Treatment with NSC23766, a selective inhibitor for Rac1-GEF interaction, resulted in the inactivation of Rac1. This intervention mitigated the EMT program in cancer cells carrying *CGN* c.3560C > T. Consistently, xenograft tumors with WT *CGN* showed no sensitivity to NSC23766 treatment, but NSC23766 demonstrated the capacity to attenuate tumor growth harboring c.3560C > T.

**Conclusions:**

*CGN* c.3560C > T leads to IQGAP1 overexpression, subsequently triggering Rac1-dependent EMT. Targeting activated Rac1 is a strategy to impede the advancement of cancers carrying this specific variant.

**Supplementary Information:**

The online version contains supplementary material available at 10.1186/s13046-024-02987-z.

## Background

Hereditary cancer syndrome, caused by mutations in cancer-susceptibility genes transmitted from parents to their offspring, increases the risk of certain cancers within families. With the increased application of gene sequencing techniques in clinical practice, more risk variants responsible for hereditary cancer syndrome have been identified. Mutations to pathogenic *BRCA1/2* variants, which regulate the homologous recombination DNA repair pathway, are reported to significantly elevate the risks of breast, ovarian, and other cancers [1–3]. The pathogenic *TP53*, *ATM*, *BRIP1*, *CDH1*, *CHEK2*, *NF1*, *RAD51,* and *STK11* variants are also associated with increased breast, ovarian, and uterine cancer risks [4–6].

Although several genetic variants have been reported as predisposing hereditary factors for different types of cancers, disease susceptibility genes are still largely unknown in most cases [7, 8]. Furthermore, unlike sporadic cancers arising from the accumulation of serial somatic variants, germline risk alleles often play significant roles in the pathogenesis of hereditary cancers. Hence, it is crucial to identify pathogenic variants for active surveillance and risk-reducing management of family members carrying the same mutations.

In this study, we conducted a comprehensive genetic analysis to identify novel cancer susceptibility mutations from family members with a high cancer density but without any reported pathogenic variants. After variants calling from family-based germline genome sequence data, validation in a cancer patient cohort, and mechanistic studies for tumorigenesis evaluation, the germline variant *CGN* c.3560C > T of tight junction protein, cingulin (CGN), was identified as germline cancer risk variant. This genetic variant causing the change of serine to leucine was identified as a more common allele frequency in the East Asian population but was seldom reported in other ethnicities, which can promote cancer cell behavior through overexpressed IQGAP1 and Rac1-dependent epithelial-mesenchymal transition (EMT). The cancer cell carrying *CGN* c.3560C > T leads to inferior treatment response to a standard adjuvant chemotherapy regimen, oxaliplatin, in colorectal cancer either from cell lines study or animal model. Importantly, blocking Rac1 activity by a specific inhibitor NSC23766 reverses the EMT program and attenuates *CGN*-mutant tumor growth. These findings suggest that *CGN* c.3560C > T is a risk allele associated with cancer progression and a potential actionable target.

## Materials and methods

Additional details are found in Supplementary Materials and Methods.

### Patient population

Ms. Lin (proband) was diagnosed with ovarian clear cell adenocarcinoma with peritoneal seeding at the age of 58, which was treated by debulking surgery and adjuvant chemotherapy with paclitaxel and carboplatin. At the age of 60, she was diagnosed with bilateral breast invasive ductal carcinoma. Treatment included bilateral mastectomy, adjuvant chemotherapy, radiotherapy, and endocrine therapy. The genetic analysis was conducted on the proband, two affected members, and two unaffected family members to identify novel cancer-predisposing variants. The validation cohort in this study included 222 patients who were treated at National Cheng Kung University Hospital, Tainan, Taiwan, for cancers of the colorectum (*n* = 122), breast (*n* = 39), ovary (*n* = 33), and endometrium (*n* = 28). No pathogenic or likely pathogenic *BRCA1/2* mutations were identified in the patients with breast, ovary, or endometrial cancers. These studies were approved by the institutional review board (IRB) of National Cheng Kung University Hospital and conducted in accordance with the Declaration of Helsinki (IRB number: A-ER-104-153 and A-ER-103-395). Written informed consents were provided by all patients.

### Library preparation and sequencing analysis

Genomic DNA (gDNA) was extracted from whole blood for the generation of a genome sequencing library and *BRCA1/2* targeted library using the TruSeq® DNA PCR-Free HT Kit (Illumina, Inc., San Diego, CA, USA) and OncomineTM BRCA Research Assay (Thermo Fisher Scientific, Waltham, MA, USA), respectively. gDNA was obtained from primary ovarian, metastatic peritoneum, and bilateral breast cancer tissues to detect somatic variants with the OncomineTM Comprehensive Assay v3 (Thermo Fisher Scientific). The resulting libraries were quantified and qualified with an InvitrogenTM QubitTM 3 Fluorometer (Thermo Fisher Scientific) and Agilent 2100 Bioanalyzer system (Agilent Technologies, Inc., Santa Clara, CA, USA), respectively. The libraries were then used for massively parallel sequencing analysis with various average sequencing depths of 30× for genome sequencing, 500× for the BRCA1/2 assay, and 1500× for the OncomineTM Comprehensive Assay v3.

### *BRCA1* and *BRCA2* variants

The proband’s family’s *BRCA1* and *BRCA2* mutational status were determined with the OncomineTM BRCA Research Assay. The variants were identified using Ion ReporterTM Software v5.10 with the OncomineTM BRCA Research Germline workflow.

### Genetic variants detection

The sequencing reads were aligned to the hg19 reference genome (https://www.ncbi.nlm.nih.gov/assembly/GCF_000001405.13/) using the Burrows-Wheeler Aligner’s maximal exact matches software package (v0.7.13; http://bio-bwa.sourceforge.net/). For detection of germline variants, sequencing reads were applied to base quality score recalibration, indel realignment, duplicate removal, as well as SNP/INDEL discovery. The genotypes were determined using standard hard filtering parameters or variant quality score recalibration according to the GATK Best Practices recommendations. For the identification of somatic variants, alternative reads were calculated with Samtools v1.3.1 mpileup to obtain the variant allele frequency in formalin-fixed and paraffin-embedded tissue specimens.

### Variant selection

Genetic variants among the proband’s family that differed from the hg19 reference genome were identified according to the following criteria: (1) a minor allelic frequency (MAF) of each variant less than 0.5% as annotated in the Taiwan biobank database to select rare variants in the Taiwan population; (2) variants located at an exon, splicing site, or other position that would cause an alteration to genetic function according to the RefGene database; (3) in consideration of the type of alteration to genetic function, only those variants leading to a change in the amino acid sequence were selected for the following filter; and (4) variants carried by at least two affected members in the proband’s family, but not by unaffected members, were selected for the validation stage.

### Candidate variant detection

One hundred one candidate variants identified from the proband’s family were used to design a targeted panel and sequence analysis. The targeted panel was composed of 101 amplicons with an average length of 148 bp. The gDNA obtained from tumor tissues was first adopted to multiplex PCR followed by constructing a massively parallel sequencing library. Following quantification and qualification, the resulting libraries were used for sequence analysis with an average sequencing depth of 1500× for each library.

### Cell line generation

Human colorectal cancer HT-29 cells and HCT-116 cells were obtained from the ATCC and maintained in McCoy’s 5A medium. Human endometrial cancer Ishikawa was a kind gift of Dr. Ching-Chou Tsai (Chang Gung Memorial Hospital, Kaohsiung, Taiwan) and maintained in Minimal essential medium (MEM) supplemented with 2 mM L-glutamine and contain 1.5 g/L sodium bicarbonate. Human bronchial epithelial cell 16HBE14o cells were purchased from Sigma-Aldrich (St. Louis, MO, USA) and were maintained in a MEM. All the cell lines were maintained in recommended media supplemented with 10% fetal bovine serum at 37 °C and 5% CO2. For the *CGN* knock-out cell line, the lentiviral expressing CRISPR-Cas9 vector, plentiCRISPRv1.4 generated by the GenScript Biotech Corporation (Piscataway, NJ, USA) was used in the study. The lentiviral vector expresses the guide RNAs (gRNAs), Cas9 protein, and puromycin resistance gene. The *CGN* gene-targeting gRNAs were designed using the software from GenScript Biotech Corporation. DNA oligonucleotides for the gRNAs and reverse complement sequence plus adapters needed for ligation were synthesized and cloned into the plentiCRISPRv1 vector. We also scrambled the *CGN* gRNAs sequence and cloned this ligated oligo into the same vector to serve as a control for characterization experiments. Different clones were sequenced and confirmed by capillary resequencing of the plentiCRISPRv1 constructs. *CGN* KO cell lines were followed by selection for stable integration using Hygromycin B (Thermo Fisher Scientific, USA). The pcDNA3.1+/C-(K)-DYK, pcDNA3.1 + N-eGFP and pcDNA3.1-CGN-eGFP were purchased from GenScript Biotech Corporation. The pAAV-G-MSCV-2A-Luc-Blank luciferase vectors were obtained from abmgood (Richmond, Canada). *CGN* c.3560C > T was generated with a site-directed mutagenesis kit (Welgene Biotech Co., Ltd., Taipei City, Taiwan). Plasmid vectors for transfection were prepared using DNA Midiprep Kits (Qiagen, Hilden, Germany) and mutations were confirmed by DNA sequencing. The cells were transfected with plasmids with jetPRIME® Transfection Reagent (Polyplus SA, Illkirch, France). At the 24th hour after transfection, GFP^+^ single cells were sorted into 96-well plates using the MoFlo® XDP cell sorter (Beckman Coulter, California, USA), and clonal lines were expanded and followed by selection for stable integration using G418 (Thermo Fisher Scientific). Different clones of cell lines were also confirmed by Western blot analysis. All cell lines have been routinely confirmed that they were mycoplasma-free.

### Animal model and IVIS system

The animal experiments were approved by the Institutional Animal Care and Use Committee (IACUC) of National Cheng Kung University (approval no. 110221, 2021/04/21) and followed the ethical guidelines. The non-obese-diabetic/severe combined immunodeficiency (NOD/SCID) mice (7–8-week-old, male, BioLasco, Taiwan) were used. Mice were housed in individually ventilated cages (3–5 mice per cage) with enrichment in temperature-controlled rooms with access to water and food ad libitum. For the orthotopic colorectal xenograft tumor model, mice were anesthetized (2% isoflurane in oxygen (2 l/min)) and randomized (blocked by cage) to receive HT-29-*CGN* WT-Luc cells or HT- 29-*CGN* S1187L-Luc cells (suspended in Matrigel, 50 μl of volume) inoculation into the cecum after the laparotomy [9]. Animal weight and condition were monitored daily. Tumor-bearing mice were randomized (blocked by cage) to receive saline, oxaliplatin, NSC23766, or NSC23766 + oxaliplatin treatment at day 7 post tumor cell implantation (NSC23766 10 mg/kg in PBS every 3 days i.p., and oxaliplatin 5 mg/kg once a week i.p.; for HT-29-*CGN* WT-Luc cells group, saline *n* = 6, NSC23766 *n* = 6, oxaliplatin *n* = 6; for HT-29-*CGN* S1187L-Luc cells group, saline *n* = 6, NSC23766 *n* = 6, oxaliplatin *n* = 6; total 36 mice across 12 cages). Cancer cell growth was imaged by the IVIS Imaging System (Xenogen, Alameda, CA, USA) and analyzed with Living Imaging software. Endpoint assays were conducted 4 weeks after the inoculation and mice were euthanized. The tumors and organs were removed and fixed in formalin and embedded in paraffin for analysis.

### Statistical analysis

Statistical analyses were performed by using GraphPad Prism 6. Results were shown as mean + SEM. Student *t*-test was performed to analyze the data among different groups. The Kaplan-Meier method with log-rank test was used to estimate the recurrent-free survival.

## Results

### Genome-wide sequencing analysis of the proband’s family

**Fig. 1:**
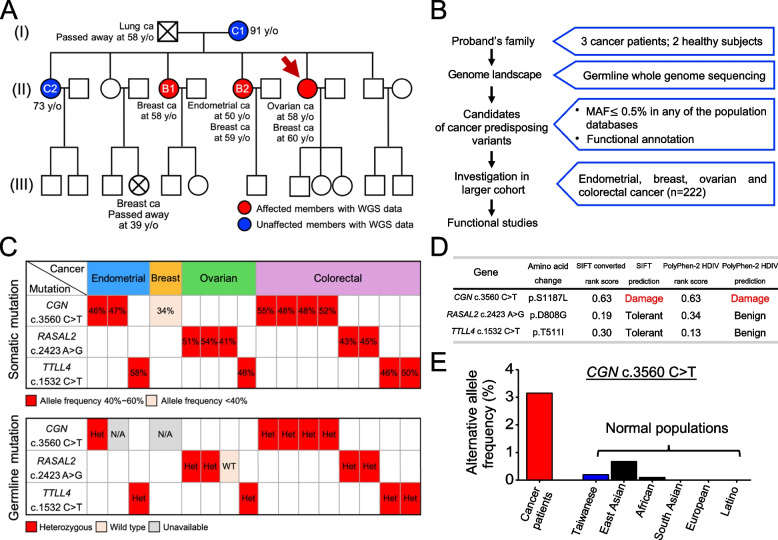
Identification of cancer-predisposing variants from the proband’s family. **A**, The pedigree of the proband. The proband is indicated by the arrow. Deceased individuals are marked with a cross. Shape symbols indicated sex: square for males and circle for females. **B**, The workflow for identifying germline cancer predisposition variants is composed of candidate variant selection, validation with cancer patient cohort, and functional studies. **C**, The germline and somatic mutational pattern of *CGN* c.3560C > T, *RASAL2* c.2423A > G, and *TTLL4* c.1532C > T in 16 patients from the validation cohort. **D**, The functional prediction of *CGN* c.3560C > T, *RASAL2* c.2423A > G, and *TTLL4* c.1532C > T based on SIFT and PolyPhen-2. **E**, The allele frequency of *CGN* c.3560C > T was compared across multiple cohorts, including the cancer patient cohort, normal Taiwanese population, and various ethnic groups enlisted in the Genome Aggregation Database (gnomAD). MAF, minor allelic frequency; Ca, cancer; Het, heterozygous; N/A, non-available; WT, wild-type; T, tolerated; D, damaging; B, benign

Ms. Lin (proband) was diagnosed with ovarian cancer and peritoneal seeding at the age of 58 and bilateral breast cancer at the age of 60 (Supplementary Fig. S1). Her father died of lung adenocarcinoma, and three members of her family were diagnosed with breast cancer, endometrial cancer, or both (Fig. 1A). To identify the germline cancer risk allele in this family, we performed a comprehensive genetic analysis. First, the four tumor tissues from the proband, including primary ovarian cancer, a metastatic peritoneal lesion of ovarian cancer, and bilateral breast cancers, were adapted for deep-targeted sequencing with a cancer panel. However, no pathogenic variants were identified (Supplementary Table S1). Second, gDNA was isolated from whole-blood samples of the proband and her two older sisters with cancer (*B1* and *B2* in Fig. 1A) underwent *BRCA1/2* analysis and whole-genome sequencing (WGS). The *BRCA1/2* variants identified from these three affected members were all benign (Supplementary Table S2). In addition, there were no pathogenic or likely pathogenic variants identified by germline WGS from these three members (Supplementary Table S3).

### *CGN* c.3560C > T is a putative germline cancer risk variant

A workflow was designed to identify germline cancer risk variants in this family. First, we collected the germline WGS data from three affected (the proband, *B1*, and *B2* in Fig. 1A) and two unaffected family members, including the mother and oldest sister of the proband (*C1* and *C2* in Fig. 1A). Next, we selected the candidate variants existing in at least two affected, but none of the unaffected members. Additionally, the variants were filtered following the criteria described in Fig. 1B. Eventually, 101 candidate variants were identified (Supplementary Table S4).

The prevalence of the 101 candidate variants was further studied in a validation cohort of 222 patients with cancers, including colorectum (*n* = 122), breast (*n* = 39), ovary (*n* = 33), and endometrium (*n* = 28) (Supplementary Table S5), by massively parallel sequencing of tumor tissues. Among the 101 candidate variants, three variants were with higher prevalence in the validation cohort (Supplementary Fig. S2), including *CGN* c.3560C > T (p.Ser1187Leu), *RASAL2* c.2423A > G (p.Asp808Gly), and *TTLL4* c.1532C > T (p.Thr511Ile).

The germline and somatic mutation patterns of *CGN* c.3560C > T, *RASAL2* c.2423A > G, and *TTLL4* c.1532C > T in the 16 patients were further explored. As shown in Fig. 1C, most patients carried the identical heterozygous germline mutation as their tumor tissues. We then used Sorting Intolerant from Tolerant (SIFT) and Polymorphism Phenotyping v2 to predict the possible impact of the three variants on the structure and function of proteins. Among them, only *CGN* c.3560C > T is predicted to be damaging (Fig. 1D). Moreover, the prevalence of germline *CGN* c.3560C > T was significantly higher in the validation cohort (3.15%) as compared to the general Taiwanese population (0.2%). According to the Genome Aggregation Database (gnomAD), the MAF of the variant was 0.67% in East Asians, 0.004% in Africans, and absent in other populations (Fig. 1E). Therefore, this East Asian-dominant variant has not been reported in The Cancer Genome Atlas (TCGA) database. Based on these results, we thus proposed that *CGN* c.3560C > T might be a putative germline cancer-susceptible variant.

### *CGN* c.3560C > T induces oxaliplatin resistance and promotes tumor metastasis in cancer cells

There is limited information on *CGN* mutation in tumor biology. Cingulin is localized to the cytoplasmic region of vertebrate tight junctions, interacting with several tight junctional proteins, such as zonula occludens, actin, and myosin, and regulating signal transduction by binding to regulators of Rho family GTPases [9–12]. We then tested the hypothesis that *CGN* c.3560C > T is associated with cancer susceptibility by the model of colorectal, endometrial, or ovarian cancer utilizing surgical specimens and multiple cancer cell lines.

**Fig. 2:**
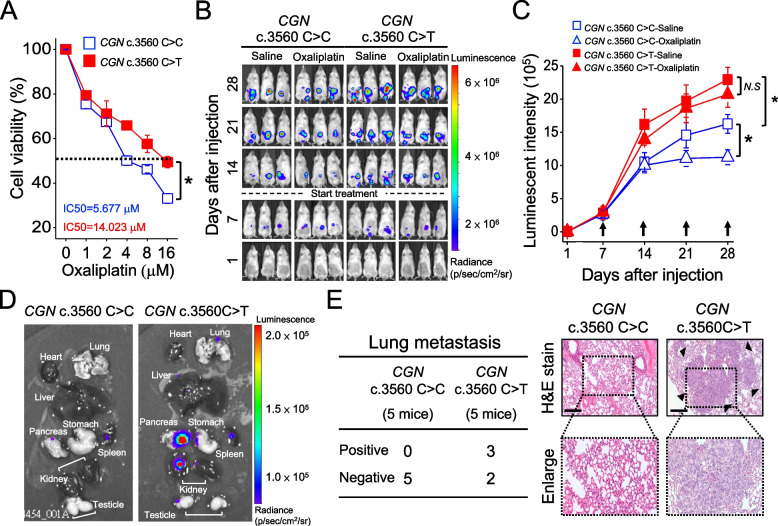
*CGN* c.3560C > T leads to oxaliplatin resistance in cancer cells. **A**, Dyk-CGN c.3560 C > C or c.3560C > T were transient transfection in HT-29 cells and treated with different concentrations of oxaliplatin (0 μM, 1 μM, 2 μM, 4 μM, 8 μM, and 16 μM) for 24 h for the MTT assay. The IC_50_ values of oxaliplatin in HT-29 cells were measured by MTT assay. Value, mean ± SEM from analysis of three different clones. **B**, Representative images showing tumor growth in Luc*-CGN* c.3560 C > C and c.3560C > T HT-29 orthotopic xenograft tumor receiving saline and oxaliplatin treatment were assessed by IVIS system. Mice were repeatedly imaged until week four after inoculation to record luminescence signals. The data were shown as radiance (photons/ sec/ cm^2^/ steradian) with a color bar. **C**, Quantitative analysis of luminescence intensity from each tumor. Mice were analyzed by optical bioluminescence imaging at 1, 7, 14, 21, and 28 days after cancer cell injection. Arrows indicate the treatment time points. Value, mean ± SEM, *n* = 6 in each group. **D**, Luciferase bioluminescence signal detection of various organs. **E**, (Upper) The frequencies of pulmonary metastasis were assessed in mice carrying Luc-*CGN* 3560 C > C or c.3560C > T HT-29 xenograft tumors at the 28th days after injection. Metastasis frequencies based on the number of mice were shown. (Lower) H&E staining results of lung tissues in *CGN* c.3560 C > C group and *CGN* c.3560 C > T group. Melanoma foci formed in the lungs were observed in *CGN* c.3560 C > T group (black arrows). Original magnification, × 10, scale bar, 100 μm. Data are presented as the mean ± SEM. *t*-test for statistical significance, *p < 0.05.; *N.S*, ≥ 0.05, not significant

To study whether *CGN* c.3560C > T affects the prognosis, we transfected Dyk-*CGN* c.3560 C > C or c.3560C > T into three different cancer cell lines, including two colorectal cancer cell lines (HT-29 and HCT-116 cells) and one endometrial cancer cell line (Ishikawa) to evaluate cell viability in the presence of chemotherapeutic agent platinum (Supplementary Fig. S3). As shown in Fig. 2A and Supplementary Fig. S4, the inhibitory effect of oxaliplatin is decreased and resulted in nearly a 3-fold resistance in all three cancer cells carrying c.3560C > T versus wild type (WT). Next, we injected luciferase (Luc)-expressing *CGN* c.3560C > C and c.3560C > T HT-29 cells into the cecal wall of NOD/SCID mice to create an orthotopic animal model. On the 28th day post-injection of cancer cells, the tumor growth, monitored by bioluminescence, was significantly promoted in *CGN*-mutant tumors compared to WT, and it cannot be suppressed by oxaliplatin treatment (Fig. 2B and C; Supplementary Fig. S5A and B). Besides, the variant induced tumor metastasis to multi-organs, which had not been observed in WT (Fig. 2D). For example, lung metastasis confirmed by bioluminescence and histologic analysis was seen in 60% (3 of 5 mice) of *CGN* c.3560C > T orthotopic xenograft but none in WT (Fig. 2E, Supplementary Fig. S5C-D). In brief, *CGN* c.3560C > T enhances tumor metastasis and is associated with decreased sensitivity to oxaliplatin treatment.

### *CGN* c.3560C > T induces the epithelial-mesenchymal transition (EMT) program and alters actin arrangement in cancer cells

**Fig. 3:**
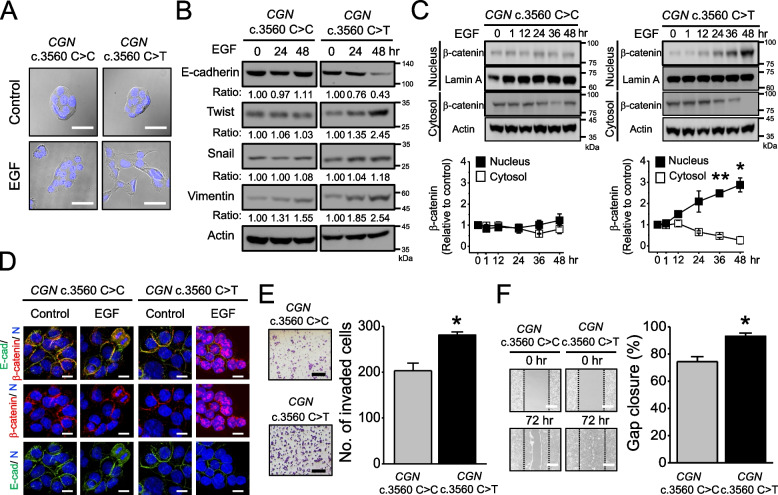
*CGN* c.3560C > T induces a mesenchymal phenotypic switch in HT-29 cells. **A**, Morphological change induced by 100 ng/ml EGF in *CGN* WT and c.3560C > T HT-29 cells. Photographs using the 40× objective. Scale bar: 20 μm. **B**, Western blot analyses of the expression of EMT markers in HT-29 cells with *CGN* WT and c.3560C > T after 100 ng/ml EGF treatment. Representative western blot from three different clones. **C**, (Upper) Western blot of the cytosol and nucleus β-catenin in HT-29 cells with *CGN* WT and c.3560C > T after 100 ng/ml EGF treatment. Lamin A and actin served as internal loading controls for cytosolic and nuclear fractions, respectively. (Lower) Densitometric quantification of the cytosol and nucleus β-catenin expression levels. Value, mean ± SEM from analysis of three different clones. **D**, Representative images showing E-cadherin (E-cad) (green), β-catenin (red), and DAPI nuclei (N) staining (blue) expression using immunofluorescent assay in response to 100 ng/ml EGF stimulation for 48 h. Nuclear β-catenin accumulation can be observed after EGF stimulation for 48 h in HT-29 cells with *CGN* c.3560C > T. Scale bar: 10 μm. **E**, (Left) Transwell assay and (Right) quantitative analyses of the invasion activity of *CGN* WT and c.3560C > T HT-29 cells. Column, mean ± SEM from analysis of three different clones. Scale bar: 200 μm. **F**, (Left) Migratory activities of *CGN* WT and c.3560C > T HT-29 cells were assessed by using gap closure assay. (Right) Quantitative analyses of the migration activity of *CGN* WT and c.3560C > T HT-29 cells. Gap closure area quantified by using the ImageJ software was taken as the index of cell migration activity. Column, mean ± SEM from analysis of three different clones. Scale bar: 200 μm. Data are presented as the mean ± SEM. *t*-test for statistical significance, *p < 0.05; **p < 0.01

Interestingly, *CGN* c.3560C > T HT-29 cells underwent a morphological change into fibroblast-like cells after epidermal growth factor (EGF) stimulation, which was not observed in WT (Fig. 3A). The fibroblastic phenotype is a hallmark of EMT, an important process of tumor progression. During EMT, the loss of cell junction, including tight junction and adherens junction, allows the acquisition of needed for metastasis [13–15]. To study whether this *CGN* variant induces EMT, we examined the molecular markers of epithelial to mesenchymal gene expression switch after EGF treatment in different cancer cells carrying *CGN* WT and variant. *CGN* c.3560C > T cells showed the downregulation of epithelial marker E-cadherin, and upregulation of mesenchymal marker Vimentin as well as transcription factor Twist upon EGF stimulation. Concomitantly, EGF induced nuclear localization of β-catenin in c.3560C > T but not in WT (Fig. 3B-D and Supplementary Fig. S6A-D). These findings indicate that *CGN* c.3560C > T leads to the process from the epithelial to mesenchymal phenotype in cancer cells. Consequently, *CGN* c.3560C > T promotes cell invasion and migration (Fig. 3E-F and Supplementary Fig. S6E-F).

We further studied whether *CGN* mutation is associated with the reorganization of the actin cytoskeleton, a late step in the EMT signaling cascade for cell morphogenesis and motility [16]. To minimize the interference caused by endogenous cingulin, we used CRISPR/Cas9 genome editing technology to knockout *CGN* (*CGN* KO) and introduced GFP-tagged *CGN* c.3560C > C and GFP-tagged *CGN* c.3560C > T mutation into multiple clones of HT-29 colon cancer cells. As shown in the Western blotting images, endogenous cingulin displays an approximately molecular size of 150 kDa in parental cells and around 180 kDa after being rescued with GFP-tagged *CGN* c.3560C > C and GFP-tagged *CGN* c.3560C > T in multiple clones of HT-29 cells (Supplementary Fig. S7A), and the relative expression level of GFP-CGN/actin was similar in *CGN*-mutant and WT HT-29 cells (Supplementary Fig. S7B). Notably, *CGN* KO do not affect EMT program by Western blotting (Supplementary Fig. S8). We then stained the cells with phalloidin, which binds F-actin for visualizing structures. As shown in Supplementary Fig. 9A, the image captured by confocal microscopy demonstrated that *CGN* c.3560C > T leads to reorganized F-actin network from cortical thin bundles into thick contractile stress fibers in cancer cells after EGF treatment. The measured fluorescent intensity of F-actin was decreased in *CGN* c.3560C > T as compared to *CGN* WT (Supplementary Fig. S9B-C). Collectively, the rearrangement of F-actin network into contractile stress fibers can be only observed in the cancer cells harboring *CGN* c.3560C > T, but not *CGN* WT or KO.

### *CGN* c.3560C > T induces IQGAP1 overexpression

**Fig. 4:**
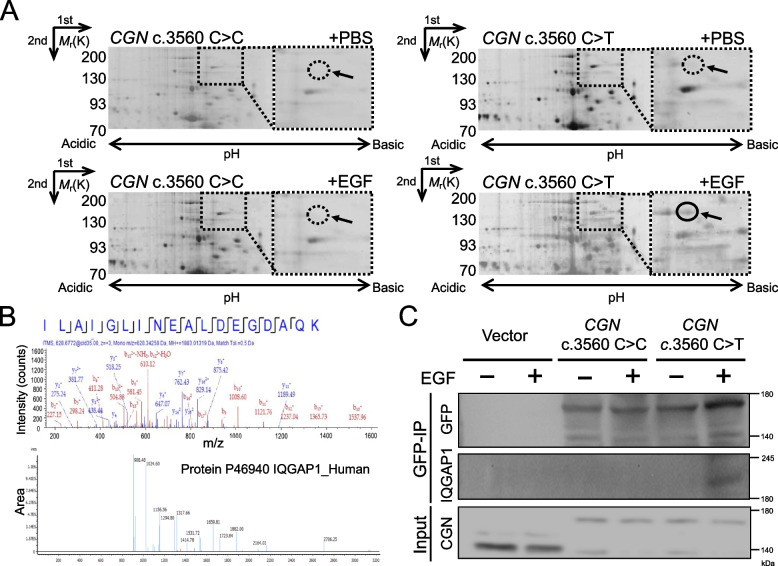
*CGN* c.3560C > T leads to overexpressed IQGAP1 identified by proteomic analysis. **A** Proteomic analysis using two-dimensional (2D) gel electrophoresis was conducted on lysates from HT-29 cells with either *CGN* c.3560 C > C or c.3560 C > T, both with and without EGF treatment over a 48-hour period. Enlarged images of specific 2D gel spots, denoted by arrows, were employed to underscore the observed variations in expression levels. A gel spot (solid circle) from *CGN* c.3560 C > T treat with EGF group was selected and followed by LC-MS/ MS analysis. **B** (Upper) Assignments to a protein in the searched database are established by correlating mass-to-charge ratio (m/z) values derived from both the Peptide Mass Fingerprinting (PMF) and the tandem mass spectrometry (MS/MS) data of the peptide ILAIGLINEALDEGDAQK. (Bottom) The peptide mapping figure and the identified sequence correspond to the protein IQGAP1. **C** The specific interaction of GFP-*CGN* WT, GFP-*CGN* c.3560 C > T, and IQGAP1 in HT-29 cells was investigated through immunoprecipitation (IP) using a GFP antibody. IP lane represents immunoprecipitation of bound eluted proteins, and input lane represents whole cell lysate. IP were analyzed by Western blotting using anti-IQGAP1 and anti-GFP antibody, and CGN expression in input was using anti-CGN antibody

To investigate the potential key elements driving EMT in *CGN* c.3560C > T cancer cells, two-dimensional (2D) gel electrophoresis-based proteomic analysis was used to visualize and compare the protein profile of *CGN* WT and *CGN* c.3560C > T with or without EGF treatment. As shown in Fig. 4A, the protein spots indicated by the arrow were only shown in *CGN* c.3560 C > T treated with the EGF group. The spots were picked and then analyzed by using LC/MS/MS (Table S6). Among the differentially expressed proteins in *CGN* c.3560 C > T treated with EGF compared to others, our focus was on spot 132, identified as IQ Motif Containing GTPase Activating Protein 1 (IQGAP1) (Fig. 4A-B and Table S6).

IQGAP1 is a scaffold protein with oncogenic potential, as it is implicated in various functions traditionally associated with cancer, including proliferation, migration, invasion, and cell-cell adhesion. Notably, IQGAP1 exhibits increased expression at both the mRNA and protein levels across various cancers that correlate with aggressiveness [17]. We further confirmed the overexpression of IQGAP1 in HT-29 and HCT-116 cells with *CGN* c.3560 C > T after EGF treatment by Western blotting (Supplementary. Fig. S10), and the direct interaction between *CGN* c.3560 C > T and IQGAP1 was demonstrated by immunoprecipitation assay (Fig. 4C).

### *CGN* c.3560C > T activates Rac1 which is regulated by overexpressed IQGAP1

IQGAP1 is a critical node within the small GTPase network and functions as a regulator of several GTPases. It functions as a regulator of various GTPases by directly binding to them and modulating their activity. Importantly, IQGAP1 not only acts as a regulator but also serves as a downstream effector of small GTPases, highlighting its multifaceted role in cellular signaling pathways [18]. Among the many binding partners interacting with IQGAP1, the Rho family is the most important small GTPase class that interacts with IQGAP1 [18–20]. We thus aim to study whether the overexpressed IQGAP1 in *CGN* c.3560 C > T cancer cells affects the activities of the Rho family.

**Fig. 5:**
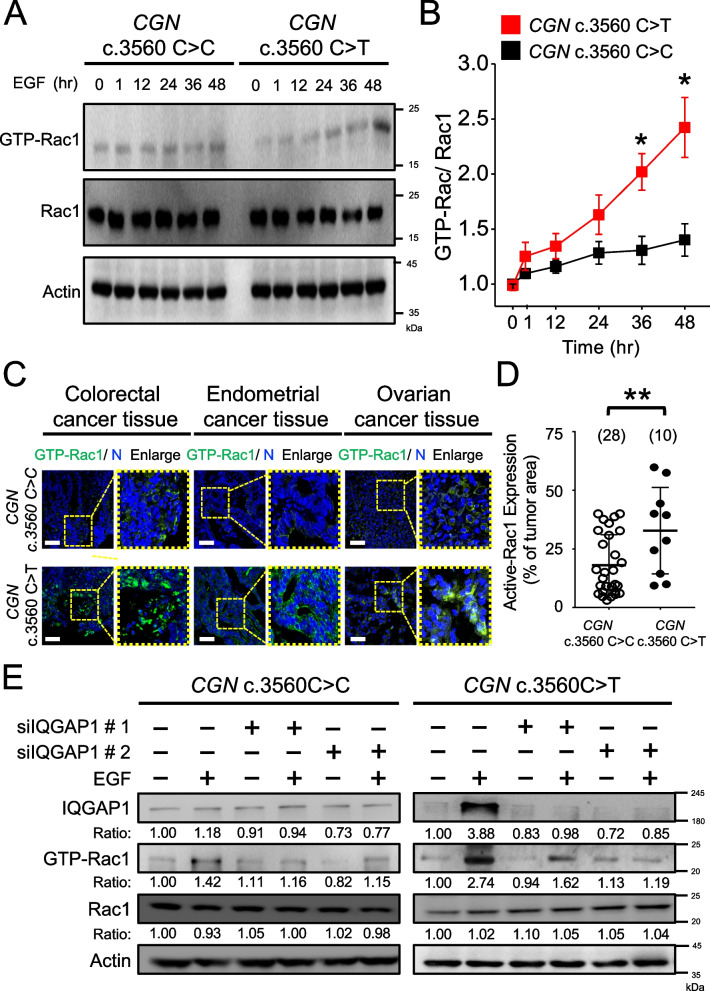
*CGN* c.3560C > T is associated with Rac1 activation. **A** Western blot of Rac1 expression level in HT-29 cells with *CGN* c.3560 C > C or c.3560C > T. **B** Densitometric quantification of GTP-Rac1 expression levels. Value, mean ± SEM from analysis of three different clones. **C** Representative confocal images of GTP-Rac1 (green) and DAPI nuclei (N) (blue) staining from 38 human tumor samples, including colorectal, ovarian, and endometrial cancers. Ten tumor samples are from patients with germline *CGN* c.3560C > T heterozygous mutation and 28 tumor samples are from patients with *CGN* c.3560C > C. Scale bar: 50 μm. **D** Quantitative analysis of Rac1 intensity from confocal images of 38 tumor samples using the TissueFAXS System. **E** Western blot showing IQGAP1, GTP-Rac1, and Rac1 protein levels. Two different IQGAP1-delivered siRNA duplexes or scramble siRNAs were treated for 24 hours followed 48 hours with or without 100 ng/ml EGF stimulus. Cell lysates were then harvested for blotting. Data are presented as the mean ± SEM. *t*-test for statistical significance. *p < 0.05, **p < 0.01

The Rho GTPase family, including Rho A, Rac1, and Cdc42, regulates actin dynamics during EMT and transcription based on cycling between inactive (GDP-bound) and active (GTP-bound) forms [21, 22]. To study the effect of the c.3560C > T variant on Rho family regulation, we compared the intensity of active and inactive Rho proteins by Western blotting. Upon EGF treatment, *CGN* c.3560C > T induced significantly higher expression of GTP-Rac1 than WT in cancer cells. On the contrary, there were no differences in RhoA and Cdc42 activity between *CGN*-mutant and WT cancer cells (Fig. 5A and B; Supplementary Fig. S11). We also employed an immunofluorescent assay to assess the expression levels of GTP-bound Rac1 in cancer cells. Notably, the activation of Rac1 was observed exclusively in cells carrying the *CGN* c.3560C > T, and not in those with WT, particularly following EGF treatment in HT-29 cells (Supplementary Fig. S12). Notably, *CGN* KO did not affect the activity of Rho family proteins (Supplementary Fig. S13). This observation underscores the specificity of the impact of the c.3560C > T variant on Rac1 activation in response to EGF stimulation.

Significantly, we have validated the overexpression of GTP-bound Rac1 in human tumor samples harboring the *CGN* c.3560C > T variant (Fig. 5C and D). A total of 38 tumor samples, spanning colorectal, endometrium, and ovarian cancers, were subjected to GTP-Rac1 staining. Importantly, GTP-Rac1 was found to be markedly upregulated in tumor tissues from patients bearing the germline heterozygous *CGN* c.3560C > T, in stark contrast to those with the c.3560C > C genotype. These findings indicate an association between *CGN* c.3560C > T and the heightened expression of GTP-Rac1, substantiating our observations from both cancer cell-line studies and clinical sample analyses.

We further delved into the regulatory relationship between the overexpressed IQGAP1 and active Rac1 in *CGN* c.3560C > T mutant cancer cells. Utilizing Western blotting, we verified the reduced expression of IQGAP1 in HT-29 cells with both *CGN* c.3560C > C and c.3560C > T genotypes after transfection with IQGAP1 siRNA. Upon treatment with EGF, we observed an elevation in both IQGAP1 expression and Rac1 activation specifically in cells with *CGN* c.3560C > T. However, following the downregulation of IQGAP1 by siRNA, the activation of Rac1 was concomitantly suppressed in *CGN* c.3560C > T cells (Fig. 5E). This finding suggests a correlation between the overexpression of IQGAP1 and the activation of Rac1 in the context of *CGN* c.3560C > T, reinforcing the regulatory role of IQGAP1 in modulating Rac1 activity in these mutant cancer cells.

### Inhibition of GTP-Rac1 suppresses in vitro and in vivo *CGN* c.3560C > T-mutant tumor growth

**Fig. 6:**
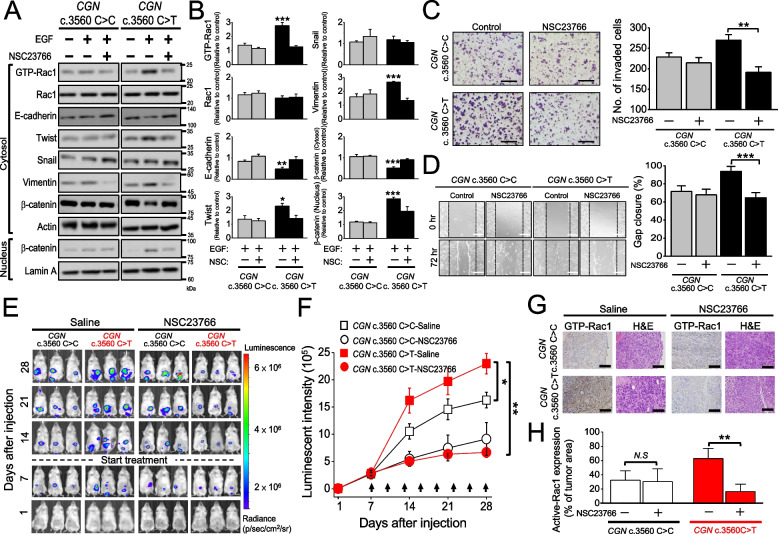
Inactivation of Rac1 reverses EMT in *CGN* c.3560C > T HT-29 cancer cells and inhibits the growth of *CGN* c.3560C > T mutant orthotopic colorectal tumors. **A** Western blot analyses of GTP-Rac1, EMT markers, and β-catenin in *CGN* WT and c.3560C > T HT-29 cells with or without NSC23766 (100 μM) and EGF (100 ng/ml) treatment. **B** Densitometric quantification of the expression level of EMT markers assessed by Western blot. Column, mean ± SEM from analysis of three different clones. **C** (Left) Transwell assay of *CGN* WT and c.3560C > T HT-29 cells with or without NSC23766 (100 μM) treatment. (Right) Quantitative analyses of the invasion activity of *CGN* WT and c.3560C > T HT-29 cells with or without NSC23766 (100 μM) treatment. Column, mean ± SEM from analysis of three different clones. Scale bar: 200 μm. **D** (Left) Migratory activities of *CGN* WT or c.3560C > T HT-29 cells with or without NSC23766 treatment assessed by using gap closure assay. (Right) Quantitative analyses of the migration activity of *CGN* WT and c.3560C > T HT-29 cells with or without NSC23766 (100 μM) treatment were assessed by using a gap closure assay. Gap closure area quantified by using the ImageJ software was taken as the index of cell migration activity. Scale bar: 200 μm. Column, mean ± SEM from analysis of three different clones. **E** Representative images showing tumor growth in *CGN* WT and c.3560C > T HT-29 orthotopic xenograft tumor receiving saline or NSC23766 treatment were assessed by IVIS system. Mice were repeatedly imaged until week four after inoculation to record luminescence signals. The data were shown as radiance (photons/ sec/ cm^2^/ steradian) with a color bar. **F** Quantitative analysis of luminescence intensity from each tumor. Mice were analyzed by optical bioluminescence imaging at 1, 7, 14, 21, and 28 days after cancer cell injection. Arrows indicate the treatment time points. Value, mean ± SEM, *n* = 6 in each group. **G** Representative image of immunohistochemistry staining for GTP-Rac1 from xenograft tumor tissues. **H** The expression level of GTP-Rac1 was detected and analyzed by HistoQuest software in tumor tissues. Column, mean ± SEM from analysis of three different clones. *t*-test for statistical significance, *p < 0.05, **p < 0.01, ***p < 0.001

Rac1 can activate actin polymerization and membrane protrusion formation resulting in cell shape changes and front-rear polarity, which is essential in EMT [23]. Our investigation has revealed a notable association between the C*GN* c.3560C > T genotype, Rac1 activation, and the EMT program in cancer cells. We aimed to explore whether this variant facilitates mesenchymal-mode movement through Rac1 activation and whether inhibiting GTP-Rac1 could impede EMT. NSC23766 is a selective inhibitor for Rac1-GEF interaction [24]. With a concentration of 100 μM, NSC23766 significantly suppressed Rac1 activation in *CGN* c.3560C > T HT-29 cells upon EGF treatment (Supplementary Fig. S14A). After inactivating Rac1 via NSC 23766 treatment, the downregulation of E-cadherin, upregulation of Vimentin and Twist, nuclear localization of β-catenin, as well as the morphological transition into fibroblast-like cells were all mitigated (Fig. 6A and B; Supplementary Fig. S14B-D). Consistently, the invasion and migration activities in *CGN* c.3560C > T cells were abolished following NSC23766 treatment (Fig. 6C and D). In Ishikawa and HCT-116 cells, the EMT caused by *CGN* mutation, which increased cell migration and invasion, was consistently reduced when NSC23766 was given (Supplementary Fig. S15). This supports the notion that the activation of Rac1 plays a pivotal role in driving the invasive and migratory capabilities of *CGN* c.3560C > T cells and inhibiting Rac1 with NSC23766 effectively mitigates these aggressive cellular behaviors.

To study the in vivo tumor growth inhibition produced by NSC23766 against *CGN* c.3560C > T-mutant tumors, we injected the mice’s cecal wall with Luc-expressing *CGN* c.3560C > C or c.3560C > T HT-29 cells and treated them with saline or NSC23766. The tumor growth was longitudinally evaluated by using IVIS (Fig. 6E). The xenograft tumors originating from WT *CGN* displayed no sensitivity to NSC23766 treatment. Conversely, NSC23766 exhibited the capacity to attenuate the growth of tumors harboring the c.3560C > T (Fig. 6F and Supplementary Fig. S16). Notably, the combination therapy involving oxaliplatin and NSC23766 did not yield a significant improvement in treatment efficacy, regardless of the *CGN* WT or c.3560C > T status. This underscores the crucial role of Rac1 inhibition specificity in addressing the growth of *CGN* c.3560C > T mutant tumors (Supplementary Fig. S17 and S18). Immunohistochemistry staining of xenograft tumors further illustrated that the elevated expression of GTP-Rac1 in *CGN* c.3560C > T tumors could be effectively suppressed following NSC23766 treatment (Fig. 6G and H). Altogether, these findings underscore that the *CGN* c.3560C > T mutation contributes to oxaliplatin resistance in colorectal cancer. However, targeting Rac1-GTP activity using the Rac1-GEF inhibitor NSC23766 can effectively suppress the EMT and subsequent aggressive cancer cell behaviors. This suggests a potential therapeutic strategy for overcoming the challenges posed by the c.3560C > T mutation in cancer.

## Discussion

This study pinpoints the c.3560C > T genetic variant within the tight junction protein CGN as a cancer susceptibility gene. A key revelation is that *CGN* c.3560C > T triggers the overexpression of IQGAP1 and activates Rac1-dependent EMT, thus promoting cancer cell behavior. The following evidence supports this conclusion. (i) *CGN* c.3560C > T was initially identified as a putative cancer-risk allele in a family with a high incidence of cancer cases, *CGN* c.3560C > T was further validated in a cohort of 222 patients with cancer during the search for novel cancer-associated variants. (ii) *CGN* c.3560C > T induces the overexpression of IQGAP1 and activates Rac1 in cancer cells, providing molecular insights into the mechanisms by which this variant influences cellular behavior. (iii) *CGN* c.3560C > T promotes EMT, demonstrating its functional impact on cellular processes and its role in driving tumor progression. Notably, this study addressed a potential targeted therapeutic strategy for cancers associated with *CGN* c.3560C > T by using the specific inhibitor NSC23766 to inactivate Rac1. This offers a avenue in the treatment of cancers characterized by defective tight junction protein CGN.

Activation of Rac1 promotes cancer cell proliferation and migration via EMT in several types of cancers, such as melanoma, colorectal, lung, and breast cancers [25–28]. Dysregulation of Rac1 has been associated with invasiveness and resistance to treatment, encompassing chemotherapy and targeted therapy [29–33]. Notably, the *Rac1* P29S “gain-of-function” mutation has been identified as a driver of melanocytes towards a mesenchymal state, inducing resistance to BRAF inhibition [31]. While *Rac1* mutations are observed, Rac1 hyperactivation resulting from overexpression, abnormal upstream inputs, and deregulated degradation is more common in human cancers [26]. Many proteins regulate the fine-tuned activity of Rho GTPase, and more than 80 GEFs and 70 GAPs for the Rho GTPase family have been identified [26, 34]. IQGAP1, in particular, has been shown to directly interact with and influence Rac1 activity, either enhancing or inhibiting its functionality [18]. For example, IQGAP1-mediated Rac1 activation occurs through the inhibition of Rac1 intrinsic GTPase activity, and the Rac1 GEF Tiam1 is recruited to IQGAP1 [35, 36]. Conversely, silencing IQGAP1 has been shown to result in high Rac1 activity during invasive cell migration, and RacGAP1 is recruited by IQGAP1 to integrin activation sites to restrain Rac1 activity [37]. In the current study, the association of *CGN* c.3560 C > T with overexpressed IQGAP1 and Rac1 activation, promoting EMT, suggests a positive correlation between IQGAP1 and Rac1 in *CGN* c.3560 C > T-mutant cancer cells. Besides its role as a regulator of GTPases, IQGAP1 is involved in the assembly of tight and adherens junctions. It modulates tight junction formation by recruiting claudin and regulates E-cadherin-mediated cell-cell adhesion and actin reorganization [38, 39]. Active Rac1 destabilizes E-cadherin-mediated cell-cell adhesion by interacting with IQGAP1, and inhibiting Rac1 activity induces increased E-cadherin-mediated cellular adhesion in certain cancer cells [40]. While both CGN and IQGAP1 localize to tight junctions and interact with other proteins to regulate tight junctional structure, the precise nature of their interaction warrants further investigation.

Several studies have suggested that Rho GTPases and their associated signaling pathways play crucial roles in cancer progression and treatment resistance, making them potential therapeutic targets [41, 42]. In our study, *CGN* c.3560C > T leads to oxaliplatin resistance in the mouse model of colon cancer. Significantly, through cancer cell line study and orthotopic xenograft animal model, a GTP-Rac1 inhibitor demonstrated the ability to suppress tumor growth specifically in *CGN* c.3560C > T mutant cancer cells, contrasting with its ineffectiveness in *CGN* WT cancer cells. Though our mechanistic studies for tumorigenesis mainly focused on the colorectal and endometrial cancer cell line, the higher expression of activated Rac1 is also demonstrated in ovarian tumor samples harboring germline *CGN* c.3560C > T. These findings address that *CGN* c.3560C > T could be a potential marker to predict cancer prognosis and identify patients who might benefit from the GTP-Rac1 inhibitor.

Approximately 5–10% of all cancers are associated with hereditary cancer syndromes [43, 44]. Identifying family members at risk for hereditary cancer syndromes is crucial for providing personalized follow-up suggestions and health management, relying on the recognition of germline-penetrant cancer-susceptibility genes. Despite considerable progress in genetic testing and understanding the clinical impact of associated genes, the scope of genetic counseling has primarily been confined to well-known genes such as *BRCA1/2, TP53, PTEN,* and other DNA mismatch repair genes. While some studies propose the use of hereditary cancer multi-gene panels [45–47]; the extensive genetic heterogeneity in cancers poses a challenge to the effectiveness of a uniform gene panel. In this study, employing tumor-normal paired analysis, pedigree-based gene mapping, cohort validation, and mechanistic studies, we identified the *CGN* c.3560C > T variant as a potential cancer-susceptibility mutation prevalent in East Asian populations. The prevalence was found to be 3.15% in the validation cohort, contrasting with 0.2% in Taiwanese individuals. Tumorigenesis usually arises from the accumulation of multiple genetic hits, dysregulated epigenetic modification, and metabolism. Notably, the presence of *CGN* c.3560C > T in only two out of three affected members in the proband’s family suggests that it might not be the sole variant responsible for tumorigenesis in this context. While our study has addressed the association between the *CGN* c.3560C > T variant and tumor progression, it is evident that additional evidence linking this variant to hereditary cancer is imperative, particularly given the current limitations in patient numbers. A more extensive examination of the variant’s prevalence among cancer patients, particularly those exhibiting a heightened familial cancer density, can yield more insights into its potential role and implications. By executing our strategic approach, we anticipate utilizing to uncover additional cancer-predisposing variants, positioning itself to substantially improve the effectiveness of cancer counseling.

## Conclusion

In conclusion, the *CGN* c.3560C > T variant leads to overexpression of IQGAP1 and activation of Rac1, correlating with metastasis and drug resistance in cancer cells. Targeting Rac1-GEF emerges as a practical and effective approach for *CGN* c.3560C > T-mutant tumors. These findings indicated that the surveillance of the mutational status of *CGN*, particularly in regions with a higher prevalence of the c.3560C > T variant. Such surveillance not only provides valuable prognostic information but also enables the tailoring of treatment strategies for patients with cancer.

### Supplementary Information

**Supplementary Material 1.**

**Supplementary Material 2.**

**Supplementary Material 3.**

**Supplementary Material 4.**

**Supplementary Material 5.**

**Supplementary Material 6.**

**Supplementary Material 7.**

**Supplementary Material 8.**

## Data Availability

The data used to support the findings of this study are available from the corresponding author upon request.

## Abbreviations

CGN: Cingulin
EMT: Epithelial-mesenchymal transition
IQGAP1: Ras GTPase-activating-like protein
IRB: Institutional review board
gDNA: Genomic DNA
MAF: Minor allelic frequency
gRNAs: Guide RNAs
KO: Knock-out
IACUC: Institutional animal care and use committee
NOD/SCID: Non-obese-diabetic/severe combined immunodeficiency
WT: Wild type
WGS: Whole-genome sequencing
SIFT: Sorting Intolerant from tolerant
TCGA: The cancer genome atlas
EGF: Epidermal growth factor
2D: Two-dimensional
MS: Mass spectrometry
LC/MS/MS: Liquid chromatography with tandem mass spectrometry
