# Supplementary material for: Tight junction protein cingulin variant is associated with cancer susceptibility by overexpressed IQGAP1 and Rac1-dependent epithelial-mesenchymal transition
Source: J Exp Clin Cancer Res. 2024 Mar 1;43:65. doi: 10.1186/s13046-024-02987-z (PMC10905802; doi:10.1186/s13046-024-02987-z)
Supplement: Supplementary file 1 — Supplementary Material 1. [file 13046_2024_2987_MOESM1_ESM.docx]

**Supplementary Information for**

**Tight junction protein cingulin variant is associated with cancer susceptibility by overexpressed IQGAP1 and Rac1-dependent epithelial-mesenchymal transition**

**This file includes:**

Supplementary Materials and Methods

Supplementary Figure S1 to S18

**Other Supplementary Materials for this manuscript include the following:**

Supplementary Tables S1 to S7

**Supplementary Materials and Methods**

**Chemicals and antibodies**

3-(4,5-Dimethyl-2-thiazolyl)-2,5-diphenyl-2H-tetrazolium Bromide, Thiazole Blue (475989, Merck) was dissolved in ddH_2_O (5 mg/ml), Epidermal Growth Factor (EGF) recombinant human protein (PHG0313, Thermo Fisher Scientific) was dissolved in phosphate-buffered saline (PBS) and diluted in a cell culture medium to a final concentration of 100 ng/ml. Two different IQGAP1 siRNA duplexes were each obtained from Santa Cruz Biotechnology (sc-35700) and Origene™ Technologies (SR305824). NSC23766 trihydrochloride (SML0952, Sigma Aldrich) was dissolved in PBS and diluted in a cell culture medium to a final concentration of 50 μM and 100 μM. The antibodies are shown in Supplementary Table S7.

**Immunoblotting and immunoprecipitation**

The cells were subjected to subcellular fractionation using a Nuclear Protein Extraction Reagent Kit (Fivephoton Biochemicals, San Diego, CA, USA) to obtain cytoplasmic and nuclear fractions. For IP, cells were washed twice with phosphate-buffered saline and scraped into Pierce™ IP lysis buffer (Thermo Fisher Scientific). Anti-GFP antibodies were coupled to M-270 epoxy Dynabeads (Thermo Fisher Scientific). IP assays were done as suggested by the Dynabeads® Antibody Coupling Kit manufacturer (Thermo Fisher Scientific). Protein concentrations were determined using a Bio-Rad protein assay (Bio-Rad Laboratories, Hercules, CA, USA). Equal amounts of protein lysates were separated by sodium dodecyl sulfate-polyacrylamide gel electrophoresis and then transferred to nitrocellulose membranes (Pall Corporation, Port Washington, NY, USA), which were blocked, incubated with the appropriate primary Ab, washed, incubated with the corresponding horseradish peroxidase (HRP)-conjugated secondary Ab (Jackson ImmunoResearch Laboratories, West Grove, PA, USA), and visualized with Western HRP substrate (GeneTex, Irvine, CA, USA). The protein bands on the immunoblots were quantified using VisionWorks LS software (UVP LLC, Upland, CA, USA).

**Immunofluorescence and scanning confocal microscope (FV-3000)**

Cells were fixed for 10 min at room temperature with 4% paraformaldehyde, washed 2–3 times with PBS, and then blocked with 3% bovine serum albumin (immunoglobulin G-free, protease-free) for 30 min at room temperature. Afterward, the cells were incubated overnight with primary antibodies (Abs) in PBS at 4°C, then rinsed at least three times in PBS with Tween 20 (PBST), incubated with secondary Abs for 1 h at room temperature, and rinsed at least 3 times in PBST. To detect the nuclei, the cells were stained with Hoechst 33258 (Invitrogen Corporation, Carlsbad, CA, USA) for 1 h at room temperature, then washed, mounted, and observed under a scanning confocal microscope (FV-3000; Olympus Corporation, Tokyo, Japan) with the fluorophores excited by a laser at 405, 488, 568, and 647 nm. To investigate the active-Rac1 expression, whole tissue was scanned with TissueGnostics GmbH FACS-like Tissue Cytometry (TissueFAXS Plus). HistoQuest software was used to analyze the positive staining area of active-Rac1 in the total tumor area. All images were collected with a confocal laser-scanning microscope. For the average actin intensity in the cell, the cell of interest was selected and the mean pixel intensity in the actin channel was measured. The pixel intensity along the line which drawn through the whole image was measured by MetaMorph software. Line plots are the average of three cells per condition.

***In vitro* functional studies**

For cell viability, HT-29 cells and HCT-116 cells were seeded in a 96-well plate at a density of 1000 cells per well and allowed to attach overnight. For each colorectal cancer cell line after oxaliplatin treatment for 24 h, 20 μL of MTT dye was added (5 mg/mL) to the wells. After 2 hours of incubation, 100 μL of dimethyl sulfoxide was added to each well to dissolve the formazan crystals and the absorbance was measured at 490 nm. IC50 concentration was determined using GraphPad Prism® software (version 5.01). For the invasion assay, 100 μl of matrigel (10%; BD Biosciences) was added to the bottom of the transwell insert. Next, 3 × 104 transfected cells were seeded onto the transwell insert along with 300 μl of medium containing 1% FBS. 1 ml of medium with 20% FBS was added to the lower part of the 24-well plate. The cells were allowed to invade or migrate for 24 h at 37°C. For the migration assay, the ibidi Culture-Insert 2 Well (ibidi GmbH, Planegg, Germany) provides two cell culture reservoirs. Cells were plated at 8 × 104 cells per well and allowed to attach overnight. The following day, culture inserts were removed, and light microscopy images were acquired. For HT-29 and HCT-116 cells, images were acquired every 24 hours. For Ishikawa cells, images were acquired every 6 hours.

**Protein extraction and in-gel digestion**

The cell pellets were lysed with lysis buffer (20 mM HEPES buffer, 0.1% SDS, 1mM EDTA) and phenylmethylsulfonyl fluoride (PMSF) on ice. The cell lysates were further sonicated in Sonicator 3000 (Misonix, Farmingdale, NY, USA) and centrifuged at 14,000. g at 4 °C for 15 min. The supernatants were transferred to a new tube, and protein concentration was determined with BCA assay (Pierce, Thermo Fisher Scientific). The excised gel spot was first de-stained and then reduced with 10 mM dithiothreitol (DTT)(Merck) at 60°C for 45 min, followed by cysteine-blocking with 55 mM iodoacetamide (IAM)(Sigma Aldrich) at 25°C for 30 min. Samples were digested with trypsin at 37°C for 16 hours. The process of protein extraction and in-gel digestion was supported by BIOTOOLS CO., LTD.

**LC-MS/MS analysis and protein identification.**

The peptides were extracted from the gel, subjected to vacuum centrifugation for drying, and reconstituted with 0.1% Formic acid before analysis. The digested peptides were diluted in HPLC buffer A (0.1% formic acid) and loaded onto a reverse-phase column (Zorbax 300SB-C18, 0.3 × 5 mm; Agilent Technologies). Subsequently, the desalted peptides were separated on a column using a multi-step gradient of HPLC buffer B (99.9% acetonitrile/0.1% formic acid) for 70 minutes with a flow rate of 0.3 μl/min. The LC apparatus was coupled with a 2D linear ion trap mass spectrometer (Orbitrap Elite ETD; Thermo Fisher Scientific), operated using Xcalibur 2.2 software (Thermo Fisher Scientific). Full-scan MS was performed in the Orbitrap over a range of 400 to 2,000 Da with a resolution of 120,000 at m/z 400. Internal calibration utilized the ion signal of protonated dodecamethylcyclohexasiloxane ion at m/z 536.165365 as a lock mass. A total of 20 data-dependent MS/MS scan events were followed by one MS scan for the 20 most abundant precursor ions identified in the preview MS scan. The m/z values selected for MS/MS were dynamically excluded for 40 seconds with a relative mass window of 15 ppm. The electrospray voltage was set to 2.0 kV, and the capillary temperature was maintained at 200°C. MS and MS/MS automatic gain control were set to 1,000 ms (full scan) and 200 ms (MS/MS) or 3 × 10^6^ ions (full scan) and 3,000 ions (MS/MS) for maximum accumulated time or ions, respectively.

Data analysis was conducted using Proteome Discoverer software (version 2.3, Thermo Fisher Scientific). MS/MS spectra were searched against the Swissprot databases with taxonomy: Yarrowia lipolytica, or a customized database using the Mascot search engine (Matrix Science, London, UK; version 2.5). For peptide identification, a mass tolerance of 10 ppm was allowed for intact peptide masses and 0.5 Da for CID fragment ions. Oxidized methionine and acetyl (protein N-terminal) were considered as variable modifications. Peptide-spectrum matches (PSMs) were then filtered based on high confidence and Mascot search engine rank 1 of peptide identification to ensure an overall false discovery rate below 0.01. Proteins with a single peptide hit were excluded. The process of LC-MS/MS analysis and protein identification was supported by BIOTOOLS CO., LTD.

**Immunofluorescence and tissue scanning**

Tumors of 16 patients with colorectal cancer, 8 with ovarian, and 14 with endometrial cancer patients treated at National Cheng Kung University Hospital were available for immunofluorescent staining. The tissue was co-stained with Hoechst 33258 to detect the nucleus. Whole tissue was scanned with TissueGnostics GmbH FACS-like Tissue Cytometry (TissueFAXS Plus). The fluorophores were excited by a laser at 405, 488, and 594 nm, respectively. HistoQuest software was used to analyze the positive staining area of Rac1-GTP in the total tumor area.

**Immunohistochemistry and H&E staining**

Immunohistochemistry (IHC) was performed on 4-μm-thick formalin-fixed paraffin-embedded sections. The antibodies used are shown in Supplementary Table S7. The procedures were done with the Bond-Max Automated IHC stainer (Leica Biosystems Newcastle Ltd, Australia) according to the following protocol. Tissues were deparaffinized with xylene and pre-treated with the Epitope Retrieval Solution 1 (citrate buffer, pH 6.0) at 100 °C for 20 min, followed by primary antibody incubated at room temperature for 30 min. Subsequently, tissues were incubated with post-primary at room temperature for 8 min using the Bond Polymer Refine Detection Kit (Leica Biosystems Newcastle Ltd, United Kingdom) and then incubated with polymer for 8 min and hydroperoxide blocking for 5 min then developed with 3,3’- diaminobenzidine chromogen for 10 min. Counterstaining was carried out with hematoxylin. For H&E staining, the sections were stained with eosin solution for 30 seconds, immersed in ethanol and xylene, and mounted for evaluation. The sections were observed under an Olympus BX51 microscope (Olympus).

**Supplementary Figures
**

**Supplementary Fig. S1. The clinical presentation and treatment course of the proband**. The proband was diagnosed with ovarian clear cell adenocarcinoma at 58 y/o, and she received surgery followed by adjuvant chemotherapy. Two years later, she was found to have bilateral breast cancer. After receiving surgery, adjuvant chemotherapy, radiotherapy, and endocrine therapy, she remained in complete remission for three years. Her four tumors, including primary ovarian cancer, metastatic peritoneal lesion of ovarian cancer, and bilateral breast cancer, were adapted to deep targeted sequencing with a cancer panel (Oncomine V3, ThermoFisher Scientific) to identify pathogenic variants. CT, chemotherapy; ER, estrogen receptor; Her2, human epidermal growth factor receptor 2; IDC, invasive ductal carcinoma; RT, radiotherapy.

**
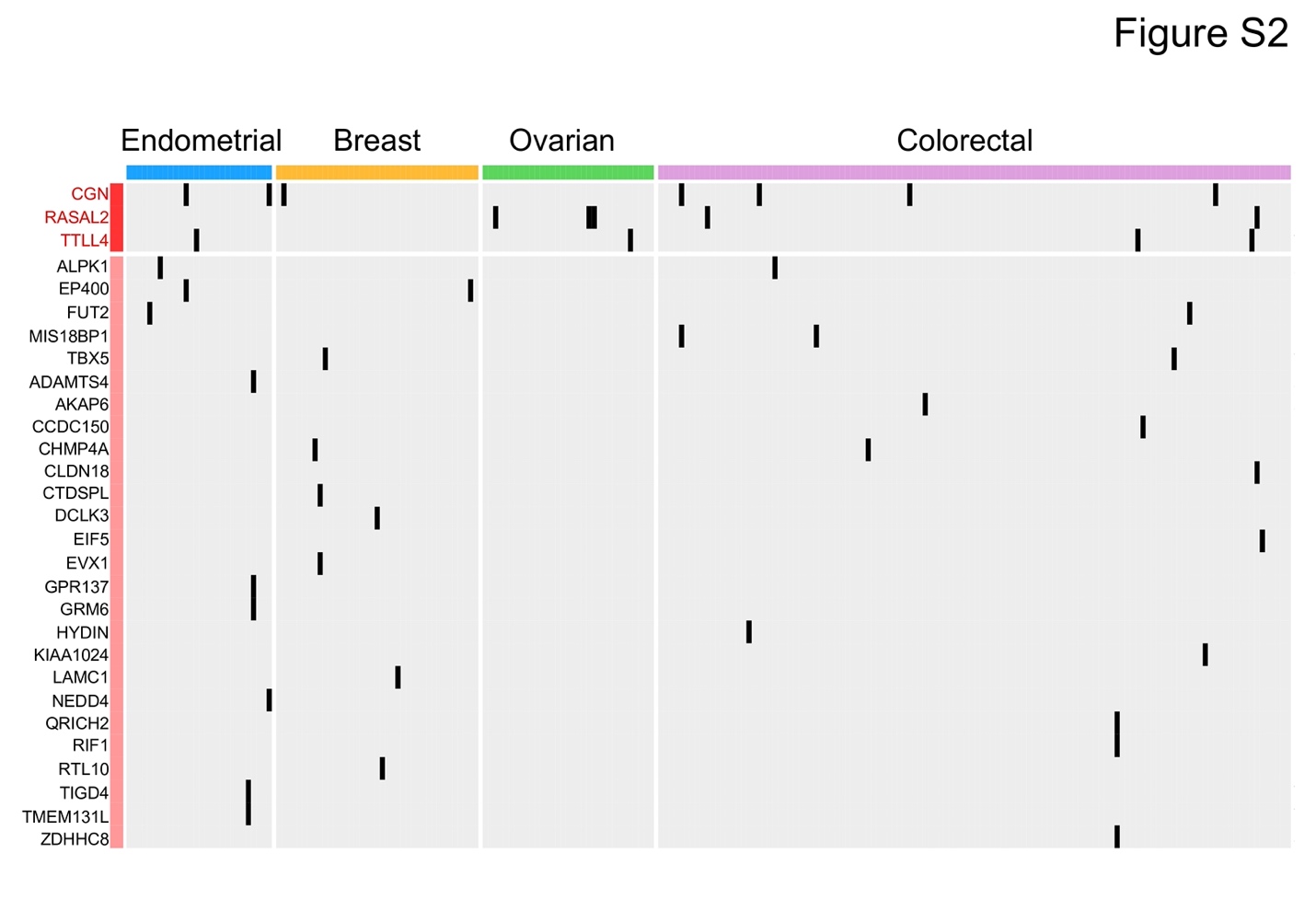
**

**Supplementary Fig S2. The prevalence of the 101 candidate variants in the validation cohort.** *CGN* c.3560C>T, *RASAL2* c.2423A>G, and *TTLL4* c.1532C>T were identified from more than two patients’ tumor tissue in the validation cohort. Each row represents a single variant, and each column indicates a single patient.

**
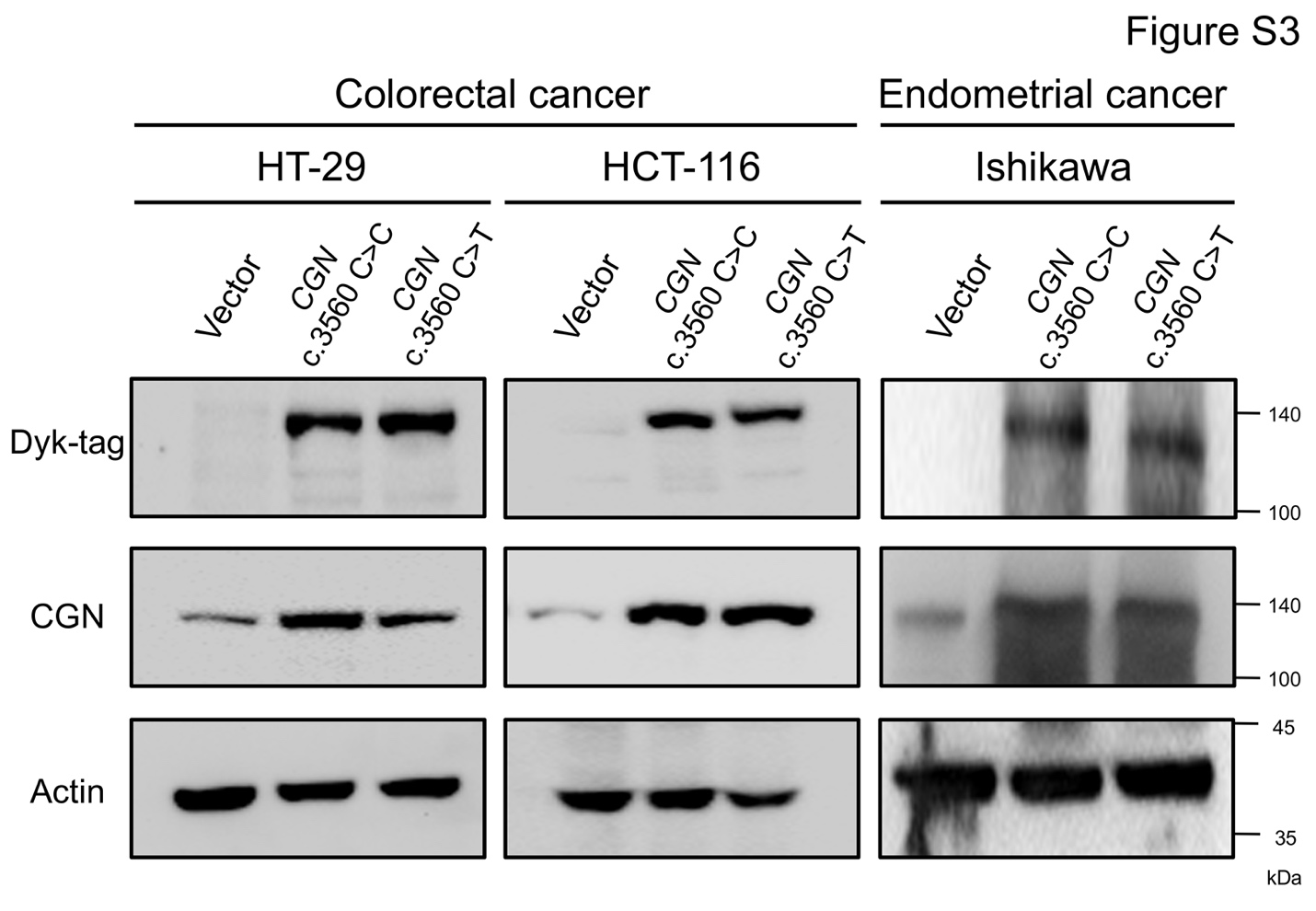
**

**Supplementary Fig. S3. Transient transfection of Dyk-tagged *CGN* c.3560C>C and c.3560C>T in HT-29 cells, HCT-116 cells and Ishikawa cells.** Total cell lysates were prepared, and equal amounts of protein were analyzed by Western blots with indicated antibodies: Dyk-tag (DYKDDDDK Tag), cingulin (CGN) and β-actin.

**
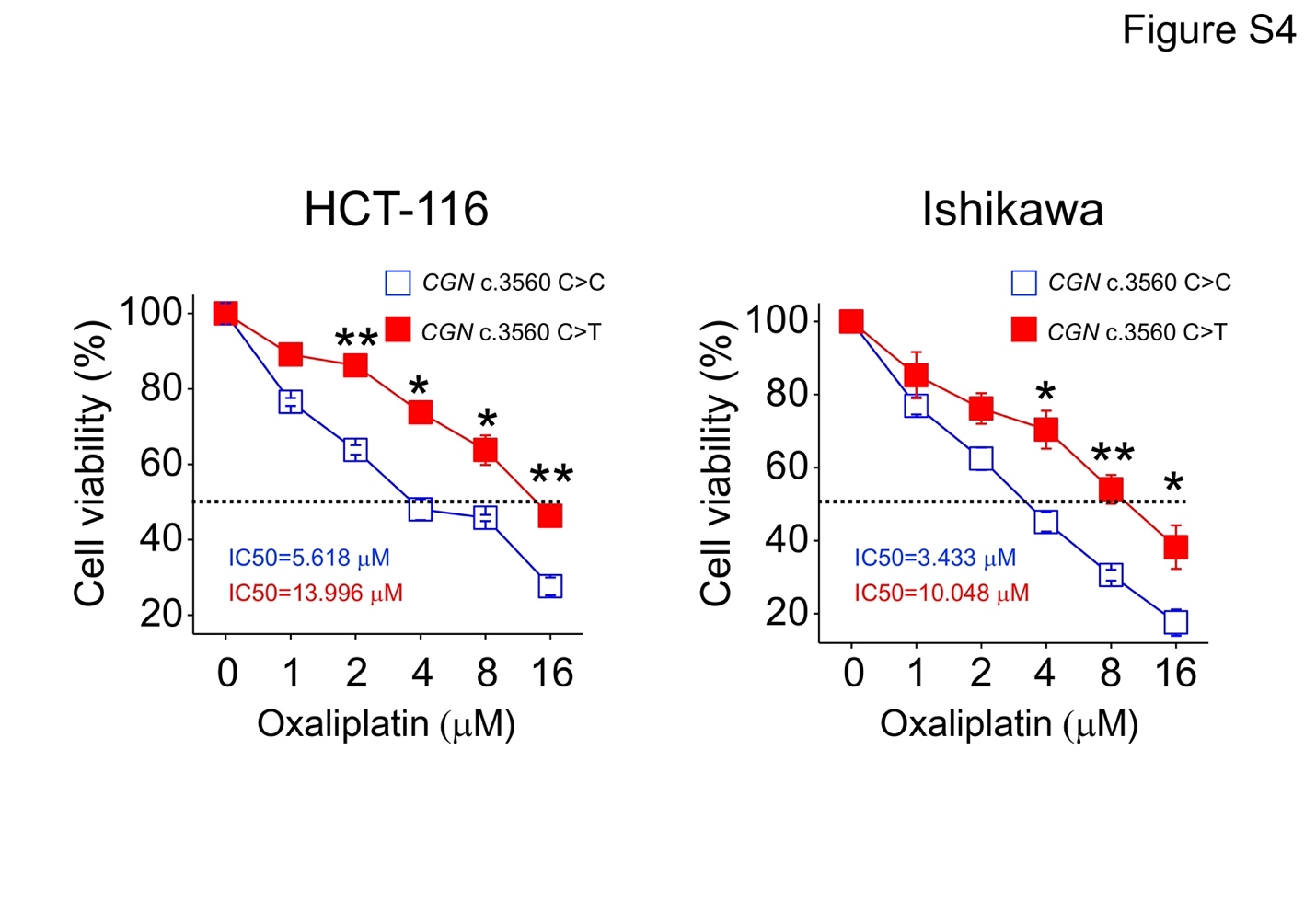
**

**Supplementary Fig. S4. *CGN* c.3560C>T leads to oxaliplatin resistance in cancer cell lines.** Dyk-*CGN* c.3560 C>C or c.3560C>T were transient transfection in HCT-116 cells and Ishikawa cells and treated with different concentrations of oxaliplatin (0 μM, 1 μM, 2 μM, 4 μM, 8 μM, and 16 μM) for 24 h for the MTT assay. The IC_50_ values of oxaliplatin in HCT-116 cells and Ishikawa cells were measured by MTT assay. Value, mean + SEM from analysis of three different clones. *t*-test for statistical significance, **p* < 0.05; ***p* < 0.01.

**Supplementary Fig. S5. *CGN* c.3560C>T promotes pulmonary metastasis in HT-29 orthotopic mouse model. A,** Luciferase bioluminescence signal detection of mice at the 28^th^ day post-orthotopic xenograft. **B,** (Upper) Representative luciferase images of intestinal tumor lesions at the 28^th^ day post-orthotopic xenograft. (Lower) Quantification of the intestinal tumor lesions (photon counts) in HT-29 orthotopic mouse model. **C,** Representative luciferase and bright field images of lungs at the 28^th^ day post-orthotopic xenograft. **D,** H&E staining of lung tissues in *CGN* c.3560 C>C group and *CGN* c.3560 C>T group. Lung metastatic lesions (black arrows) were only observed in *CGN* mutant group. Scale bar, 100 μm. Data are presented as the mean + SEM. *t*-test for statistical significance, **p* < 0.05; ***p* < 0.01; *N.S*, ≥ 0.05, not significant.


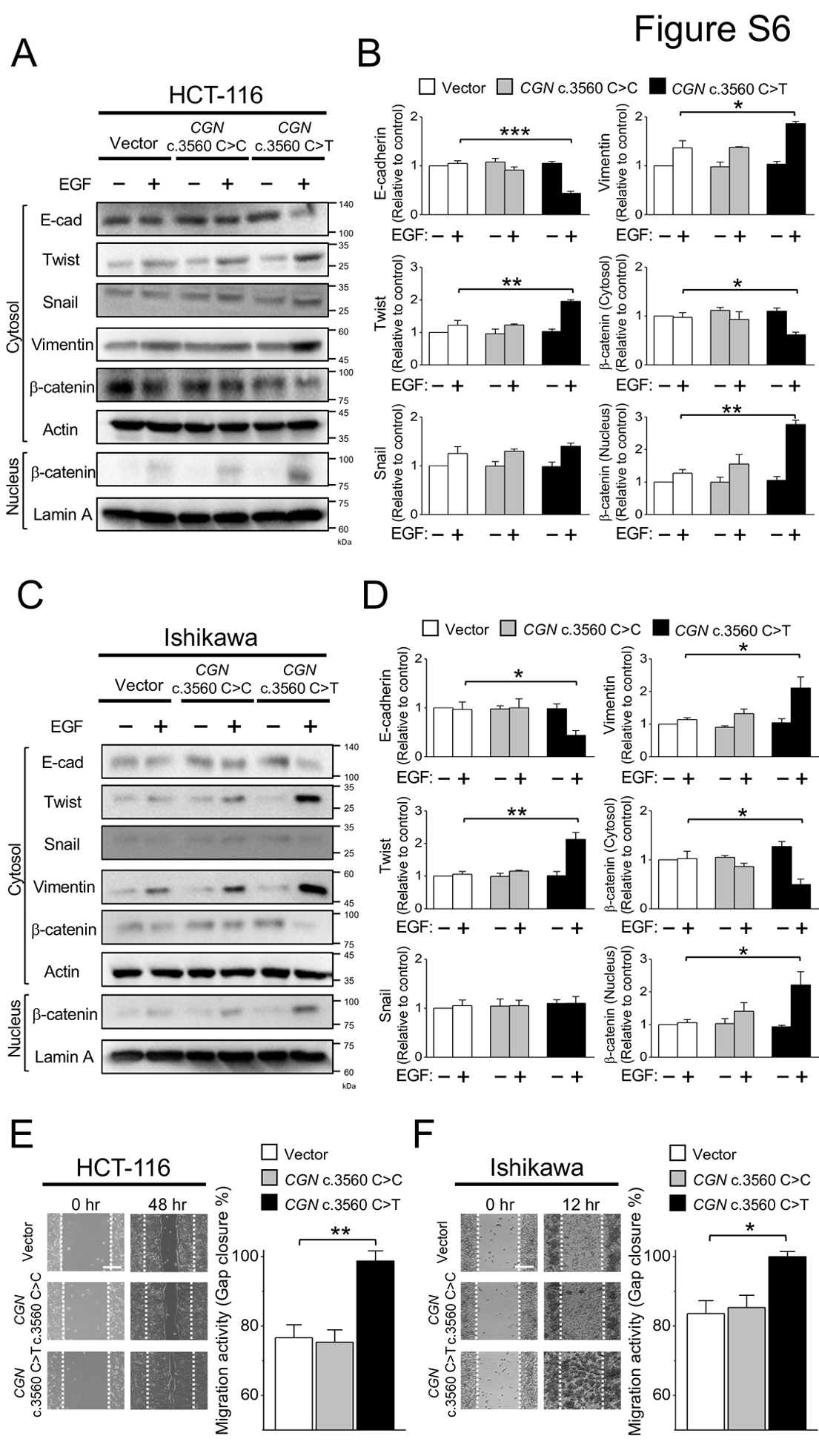


**Supplementary Fig. S6. *CGN* c.3560C>T exhibited the epithelial-mesenchymal transition (EMT) phenotype in HCT-116 cells and Ishikawa cells.**  **A, C,** Western blot analyses of the expression of EMT markers in HCT-116 cells and Ishikawa cells with *CGN* WT and c.3560C>T after 100 ng/ml EGF treatment. **B, D,** Densitometric quantification of the expression level of EMT markers assessed by Western blot. Column, mean + SEM from analysis of three different clones. **E, F** (Left) Migratory activities of *CGN* WT and c.3560C>T HCT-116 cells **(E)** and Ishikawa cells **(F)** were assessed by using gap closure assay. (Right) Quantitative analyses of the migration activity of *CGN* WT and c.3560C>T HCT-116 cells **(E)** and Ishikawa cells **(F)**. Gap closure area quantified by using the ImageJ software was taken as the index of cell migration activity. Column, mean + SEM from analysis of three different clones. Scale bar: 500 μm. Data are presented as the mean + SEM. *t*-test for statistical significance, **p* < 0.05; ***p* < 0.01; ****p* < 0.001.


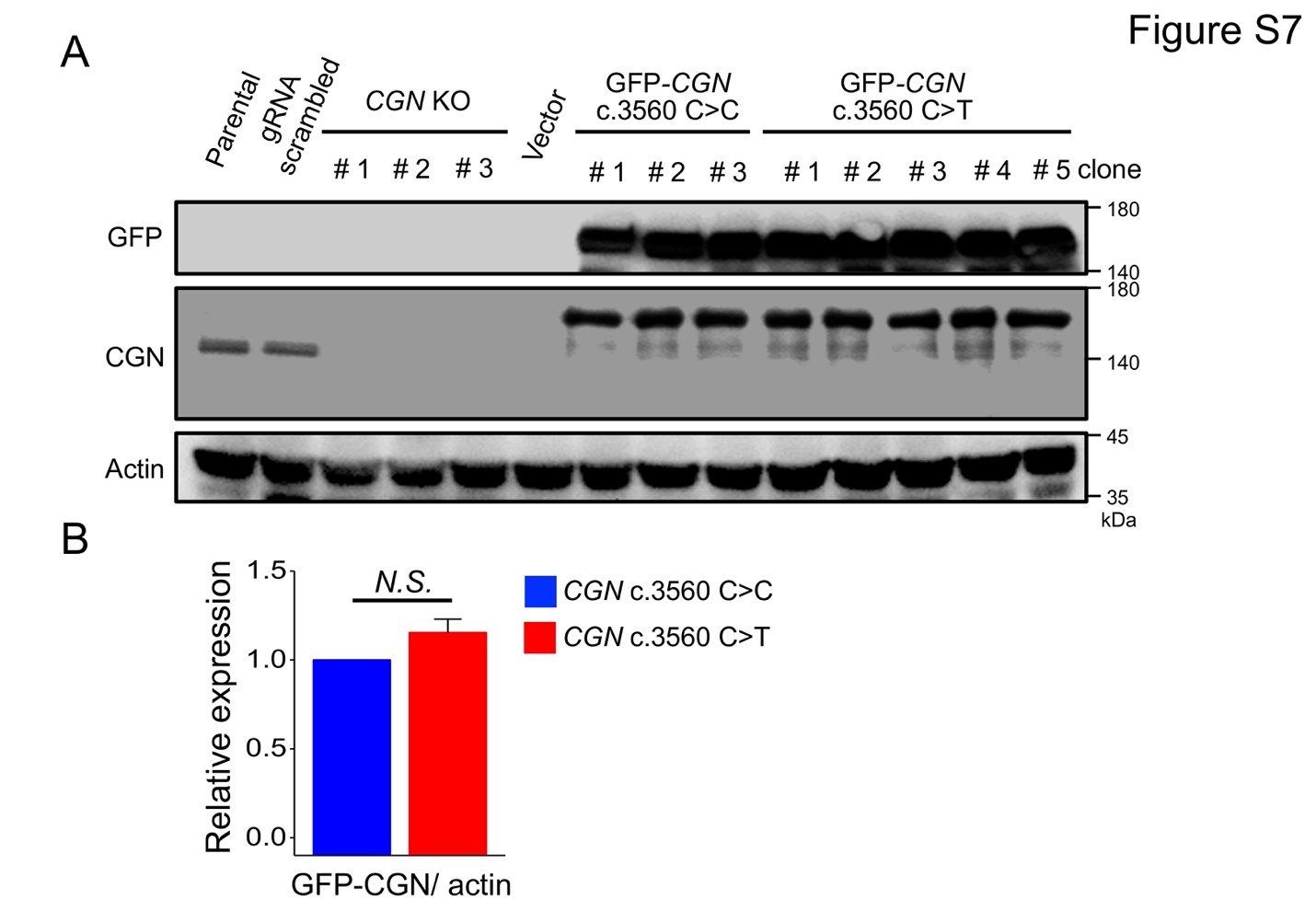


**Supplementary Fig. S7. Generation of *CGN* knockout, GFP-tagged *CGN* c.3560C>C and c.3560C>T HT-29 cells. A,** The expression level of GFP and CGN in parental, gRNA scrambled, *CGN* KO, *CGN* c.3560C>T and c.3560C>T HT-29 cells were measured by using Western blotting. **B,** Densitometric quantification of the expression level of GFP-CGN in parental, gRNA scrambled, *CGN* KO, *CGN* c.3560 C>C and c.3560C>T HT-29 cells, taking parental as 1.0. Column, mean + SEM from analysis of three different experiments. *t*-test for statistical significance, *N.S*, ≥ 0.05, not significant.


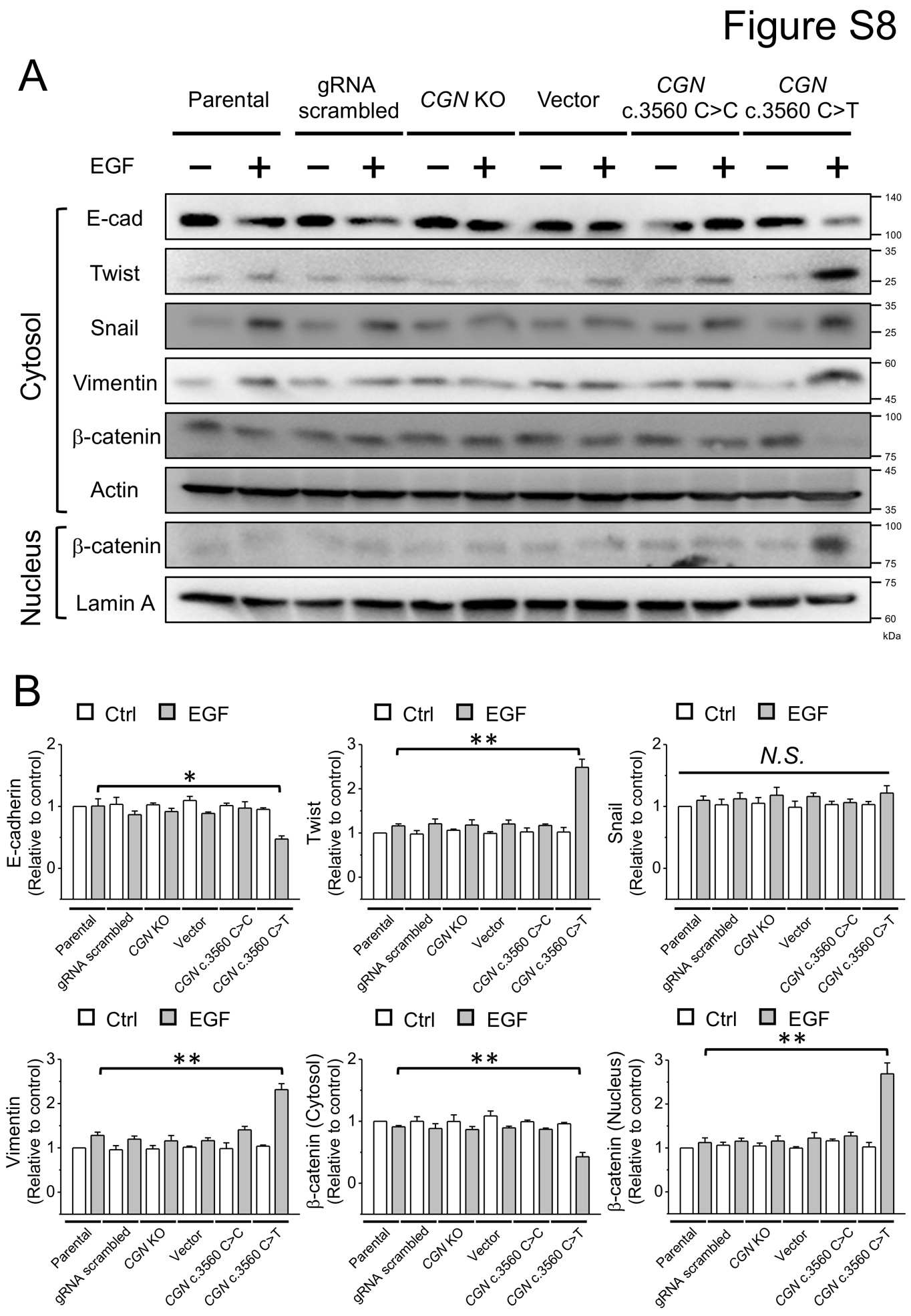


**Supplementary Fig. S8. The EMT program can be induced only in *CGN* c.3560 C>T genotype. A,** Western blot analyses of the expression of EMT markers in different clones of HT-29 cells in parental, gRNA scrambled, *CGN* KO, *CGN* c.3560C>C, and *CGN* c.3560C>T after 100 ng/ml EGF treatment. **B,** Densitometric quantification of the expression level of EMT marker in parental, gRNA scrambled, *CGN* KO, *CGN* c.3560 C>C and c.3560C>T HT-29 cells, taking parental without EGF treatment as 1.0. Column, mean + SEM from analysis of three different clones. *t*-test for statistical significance, **p* < 0.05; ***p* < 0.01; *N.S*, ≥ 0.05, not significant.

**
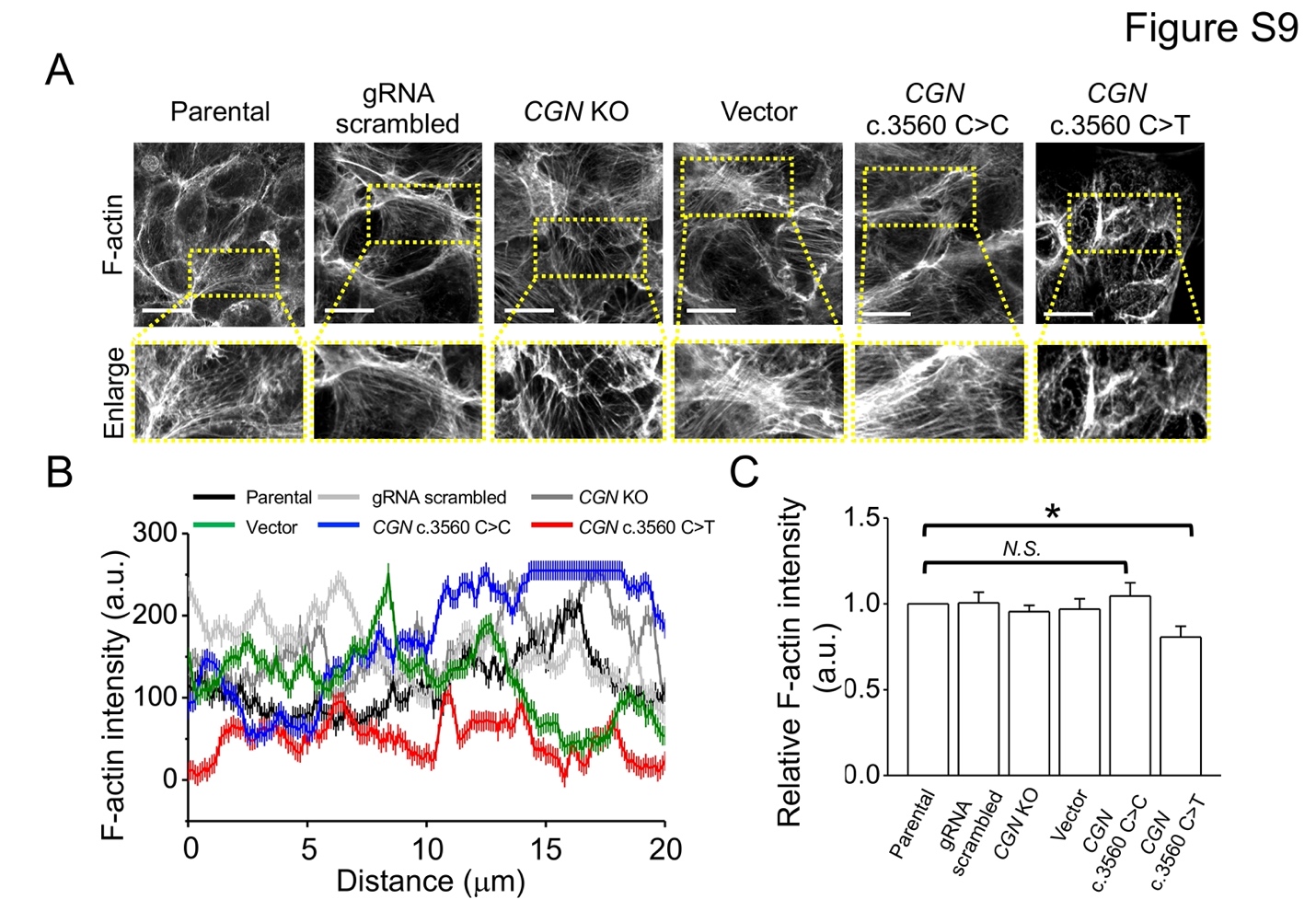
**

**Supplementary Fig. S9. The presence of *CGN* c.3560C>T induces a reorganization of the F-actin network. A,** F-actin (gray) staining expression in parental, gRNA scrambled, *CGN* KO, *CGN* c.3560C>C, and *CGN* c.3560C>T HT-29 cells after 100 ng/ml EGF treatment for 48 hours were captured by confocal microscopy. Scale bar: 10 μm. **B,** Average fluorescent intensity line profiles of F-actin measured by MetaMorph software, line plots are the average of three cells per condition. **C,** The intensity of the actin staining was measured by Image J (v1.4) software. Taking the parental group as 1.0, the staining intensity was normalized to the parental cells. Column, mean + SEM from analysis of three different clones. *t*-test for statistical significance, **p* < 0.05; *N.S*, ≥ 0.05, not significant.


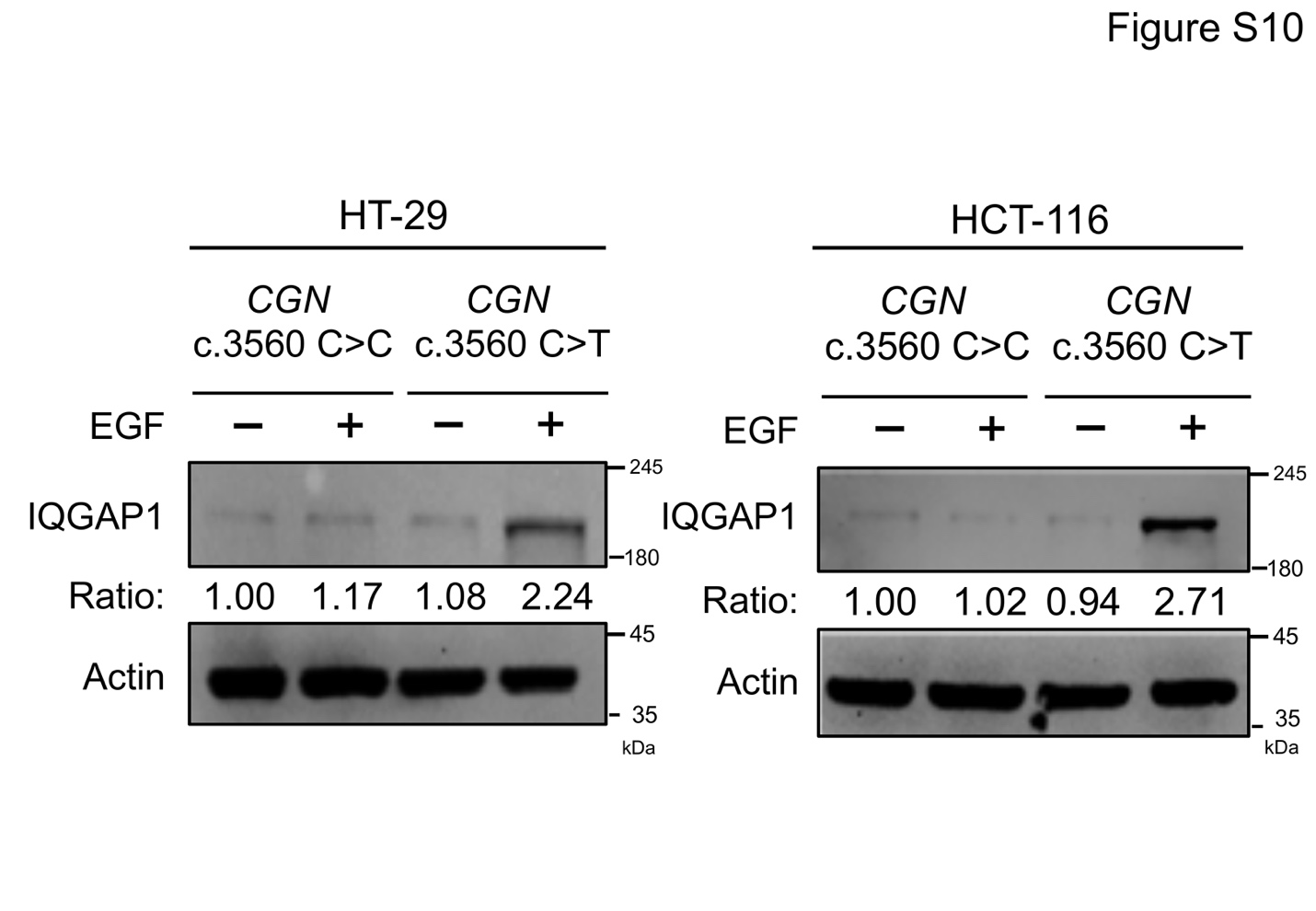


**Supplementary Fig. S10. *CGN* c.3560C>T induce IQGAP1 expression level with or without EGF treatment.** Western blot of IQGAP1 protein expression level in HT-29 cells and HCT-116 cells with or without 100 ng/ml EGF treatment after 48 hours.

**
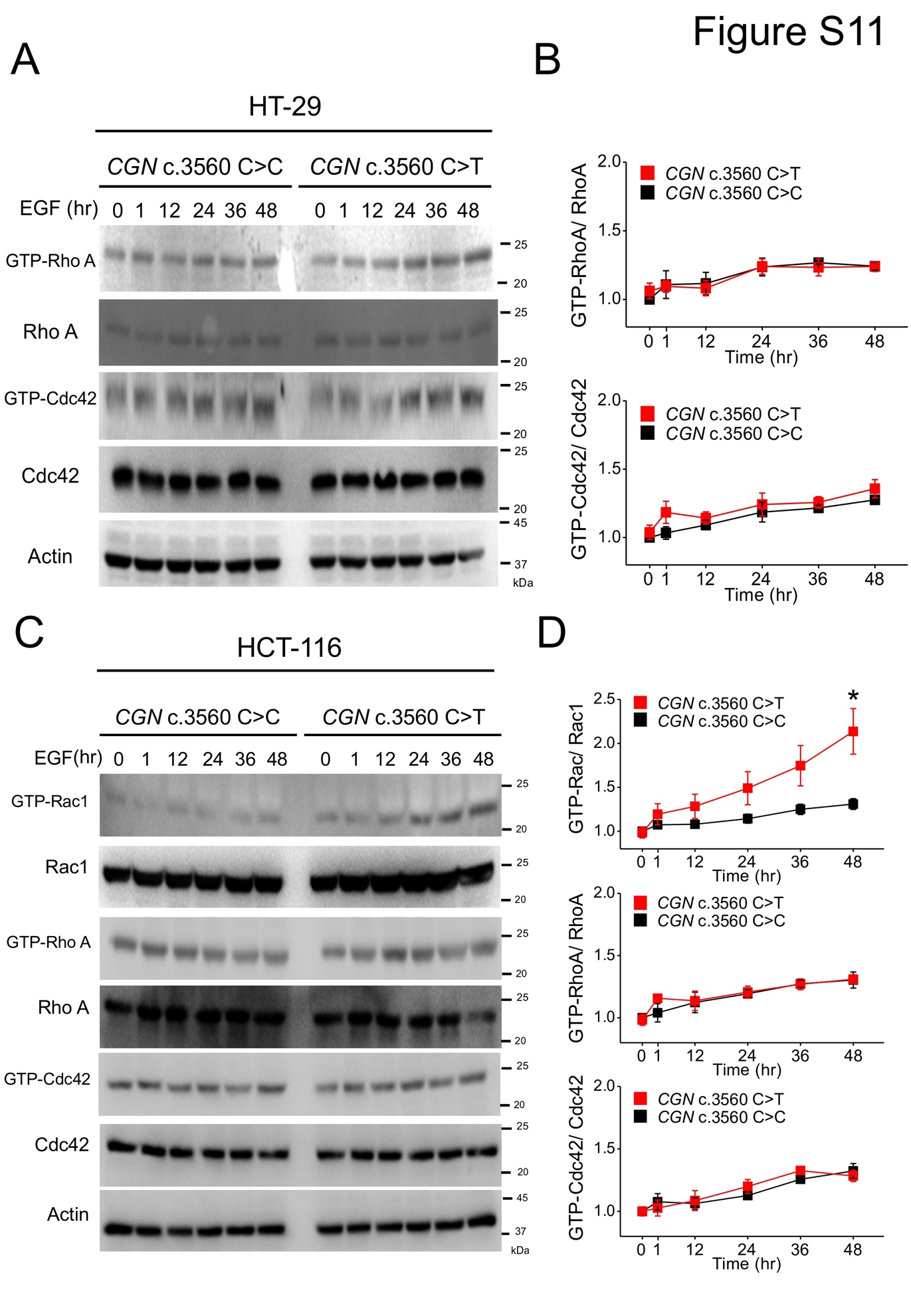
Supplementary Fig. S11. *CGN* c.3560C>T selectively activates Rac1, without influencing RhoA and Cdc42, in cancer cells.** Western blot of Rho family protein (RhoA, and Cdc42) expression level in HT-29 cells and Rho family protein (Rac1, RhoA, and Cdc42) expression level HCT-116 cells treat with 100 ng/ml EGF in each indicated time. **B, D,** Densitometric quantification of GTP-Rac1, GTP-RhoA, and GTP-Cdc42 expression levels. Value, mean + SEM from three different clones. *t*-test for statistical significance, **p* < 0.05.

**
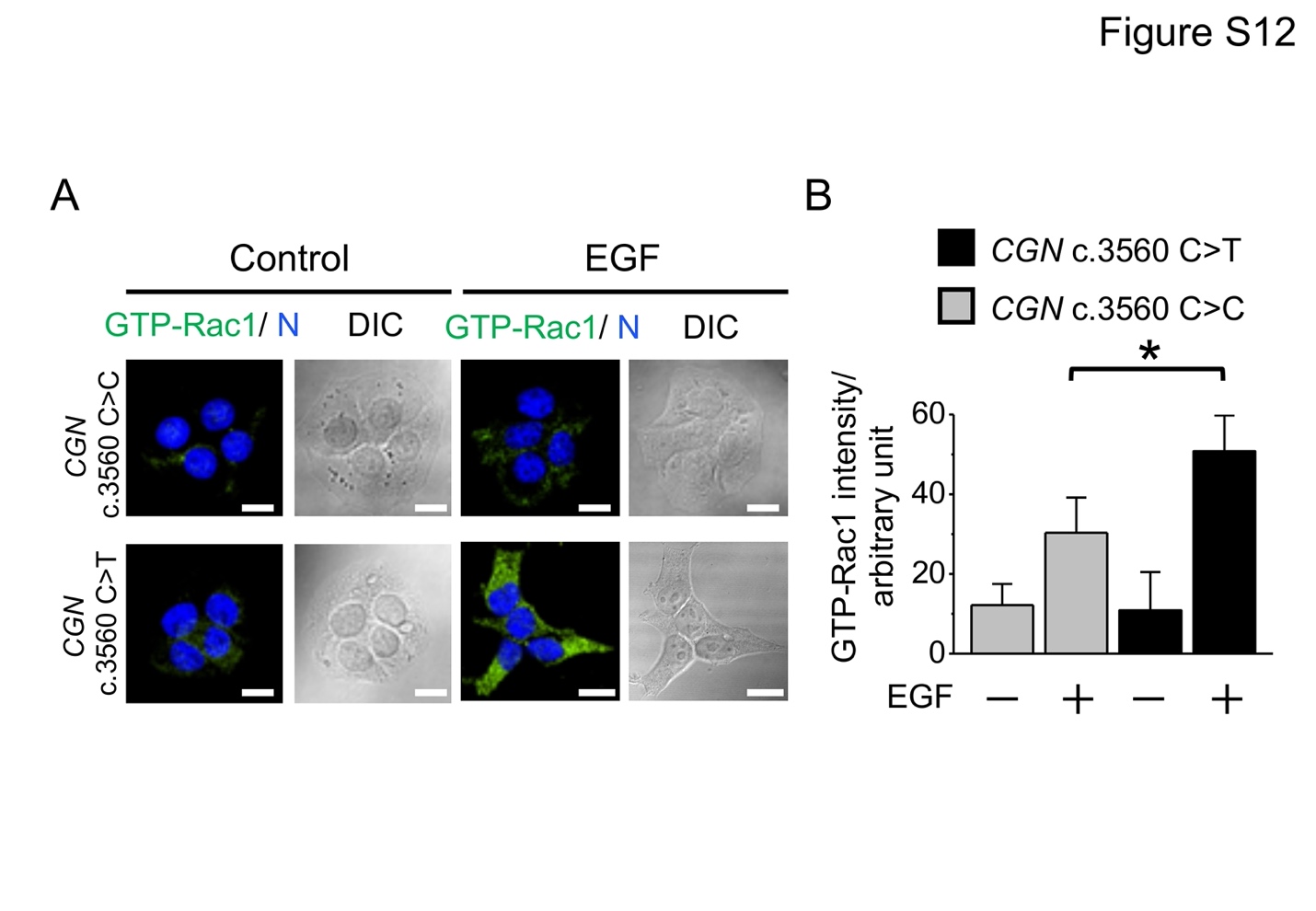
**

**Supplementary Fig. S12. *CGN* c.3560C>T triggers the activation of Rac1 in cancer cells*.* A,** Representative images showing GTP-Rac1 (green) and DAPI nuclei (N) (blue) staining expression using immunofluorescent assay in response to 100 ng/ml EGF stimulation for 48 hours. Scale bar: 10 μm. **B,** Quantitative analyses of GTP-Rac1 staining in *CGN* c.3560 C>C or c.3560C>T HT- 29 cells. Column, mean + SEM from analysis of three different clones. *t*-test for statistical significance, **p* < 0.05.

**
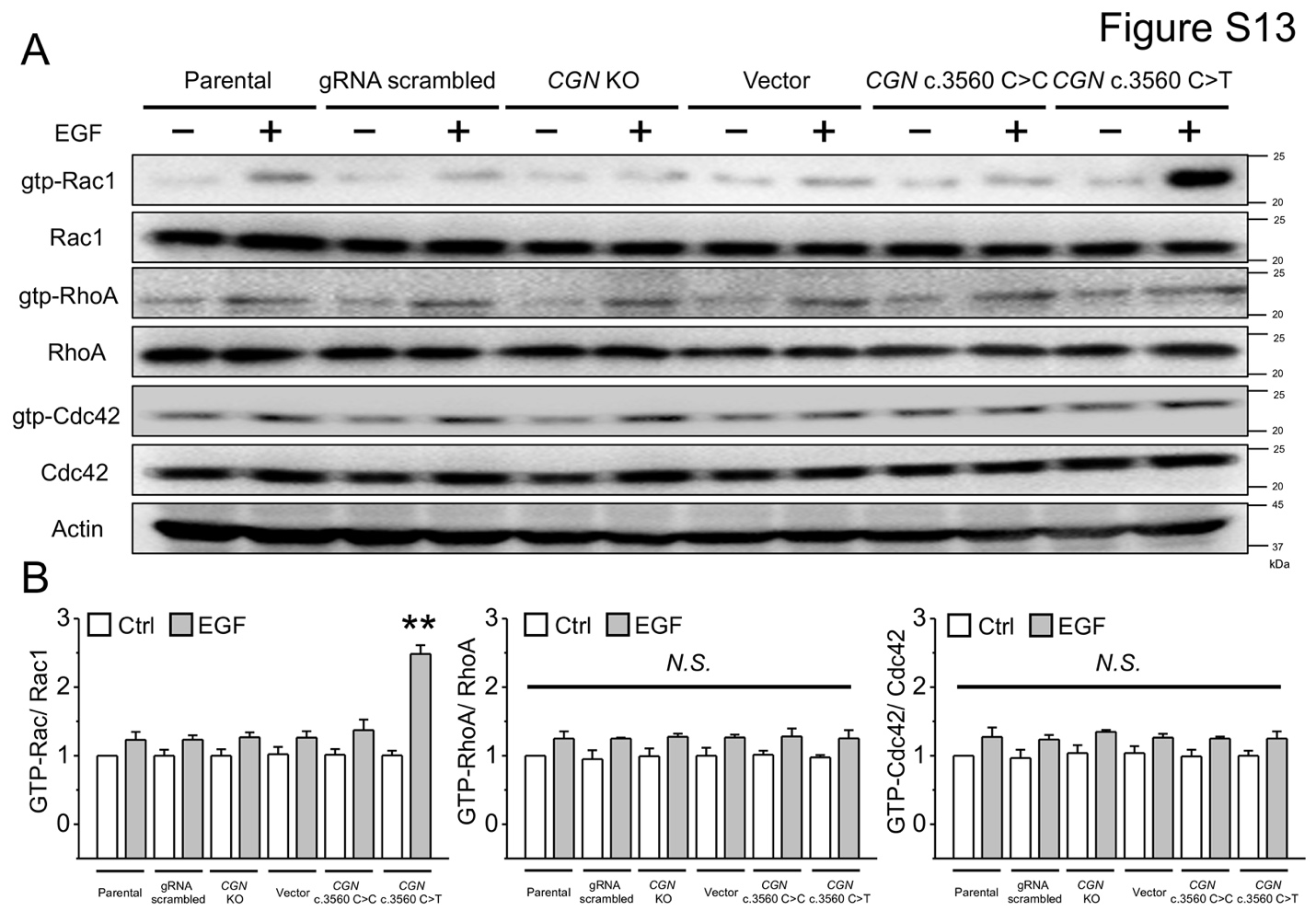
**

**Supplementary Fig. S13.** **The activation of Rac1 is specifically demonstrated in the *CGN* c.3560C>T genotype. A,** Western blot of Rho family protein (Rac1, RhoA, and Cdc42) expression level in different clones of HT-29 cells in parental, gRNA scrambled, *CGN* KO, *CGN* c.3560C>C, and *CGN* c.3560C>T after 100 ng/ml EGF treatment for 48 hours. **B,** Densitometric quantification of GTP-Rac1, GTP-RhoA, and GTP-Cdc42 expression levels. Column, mean + SEM from three different clones. *t*-test for statistical significance, ***p* < 0.01; N.S, ≥ 0.05, not significant.


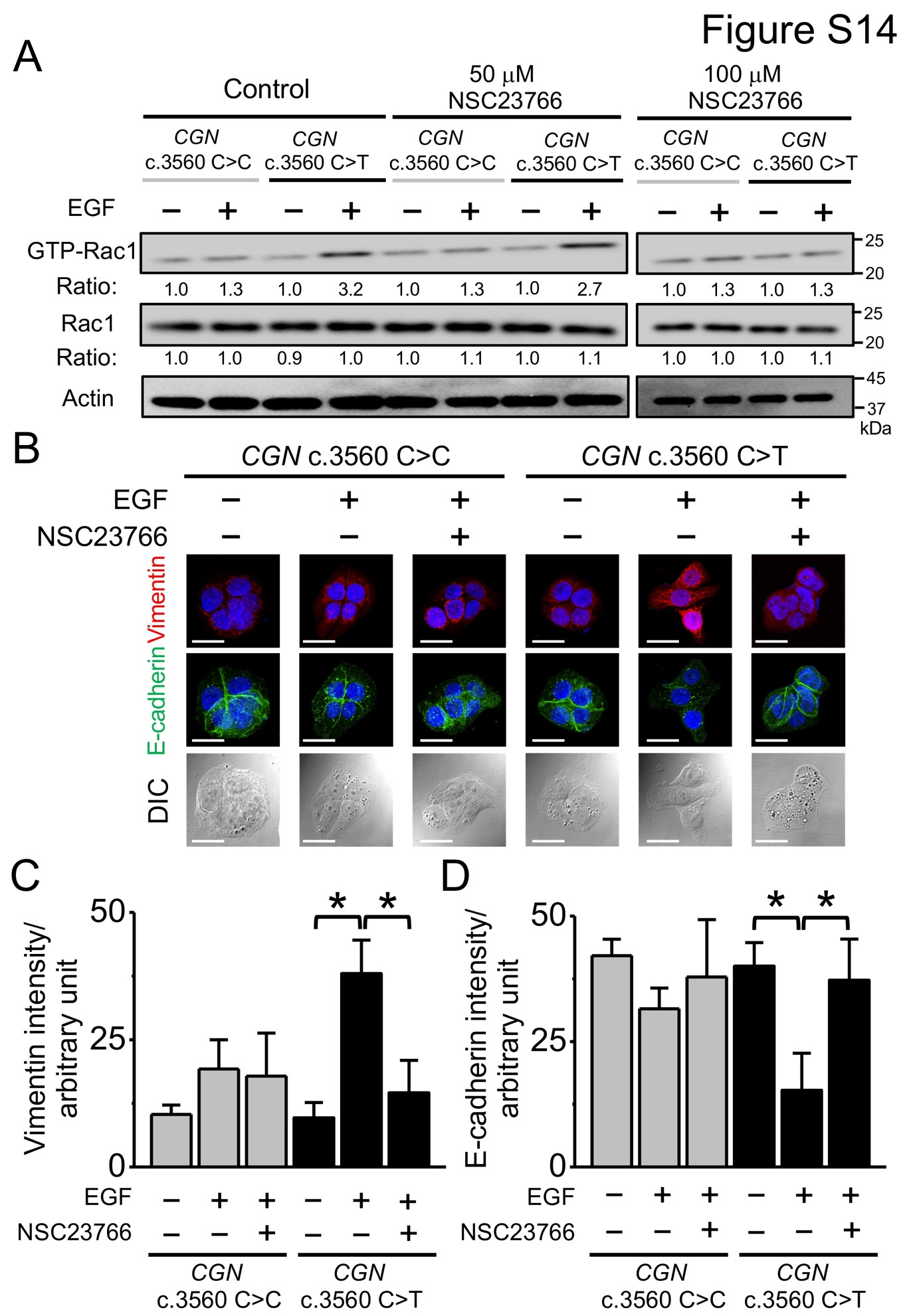


**Supplementary Fig. S14. NSC23766 inhibits *CGN* c.3560C>T-induced Rac1 activation *in vitro*. A,** The expression level of GTP-Rac1 and Rac1 in *CGN* c.3560C>C and c.3560C>T HT-29 cells with or without NSC23766 treatment at different dosage levels (50 μM and 100 μM) were measured by using Western blotting. **B,** Representative images showing Vimentin (red), E-cadherin (green), and DAPI nuclei (blue) staining expression by immunofluorescent assay in response to 100 ng/ml EGF with or without 100 μM NSC23766. Scale bar, 20 μm. **C** and **D,** Quantitative analyses of Vimentin **(C)** and E-cadherin **(D)** staining in *CGN* c.3560C>C and c.3560C>T mutation HT-29 cells with or without 100 μM NSC23766 treatment. Column, mean + SEM from three different clones. *t*-test for statistical significance, **p* < 0.05.


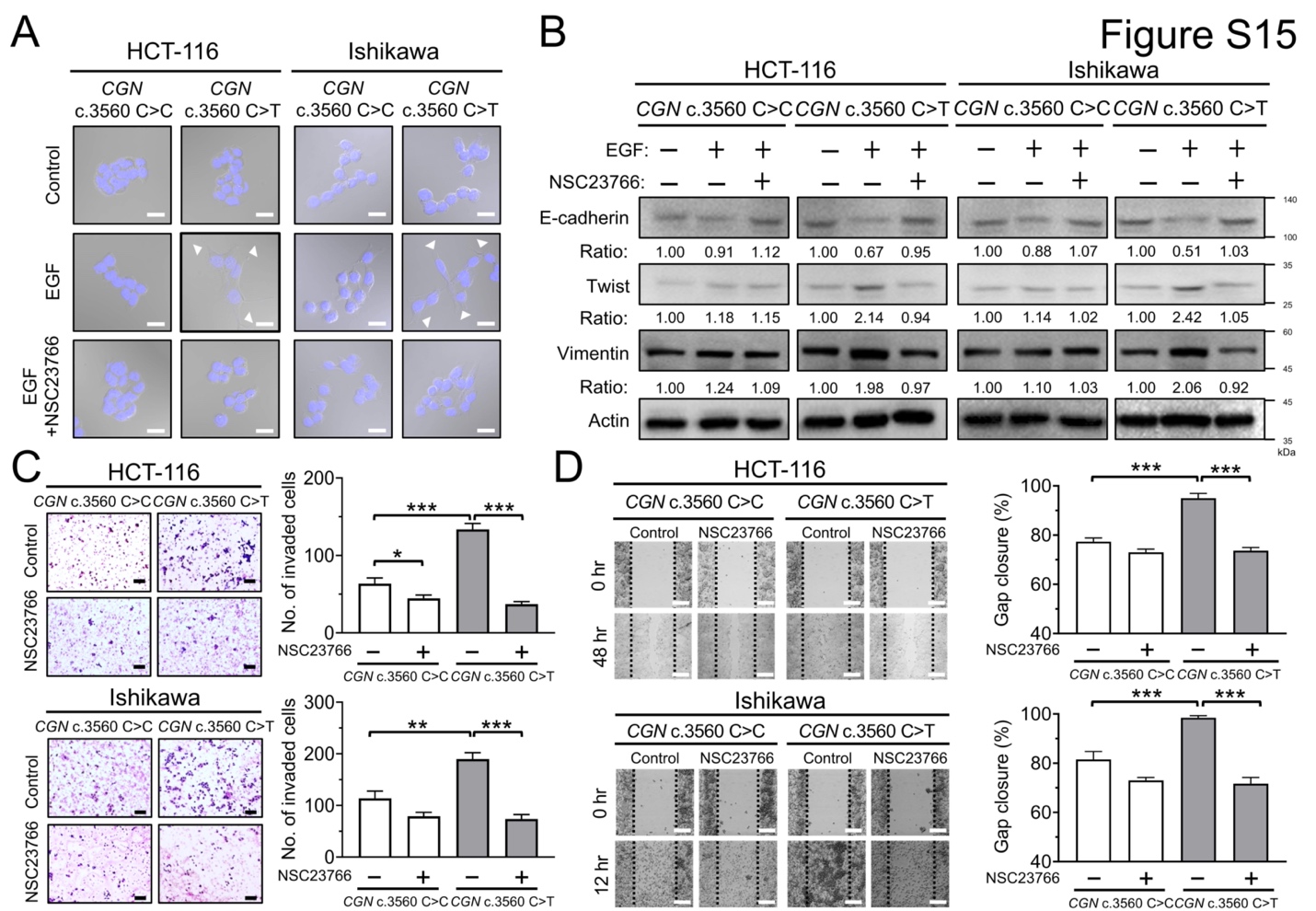

**Supplementary Fig. S15. NSC23766 suppresses *CGN* c.3560C>T-induced EMT in HCT-116 and Ishikawa cells. A,** Morphological changes observed in *CGN* WT and c.3560C>T HCT-116 and Ishikawa cells following exposure to 100 ng/ml EGF, both in the presence and absence of 100 μM NSC23766. Photographs using the 40× objective. Scale bar: 20 μm. **B,** Western blot analyses were performed to evaluate the expression of EMT markers in *CGN* WT, c.3560C>T HCT-116, and Ishikawa cells under diverse conditions, encompassing treatments with NSC23766 (100 μM) and EGF (100 ng/ml). **C,** Representative images of transwell invasion assay and bar graphs illustrating the mean account of cells with or without NSC23766 (100 μM). Column, mean + SEM from analysis of three different clones, n=9 in each group. Scale bar: 100 μm. **D,** Migratory capabilities of cells, both with and without NSC23766, were evaluated using a gap closure assay. Bar graphs were generated to visually represent the analyses of migration activity, specifically comparing cells with or without NSC23766 (100 μM). Gap closure area quantified by the ImageJ software was taken as the index of cell migration activity. Scale bar: 200 μm. Column, mean + SEM from analysis of three different clones, n=6 in each group. *t*-test for statistical significance, **p* < 0.05; ***p* < 0.01; ****p* < 0.01.


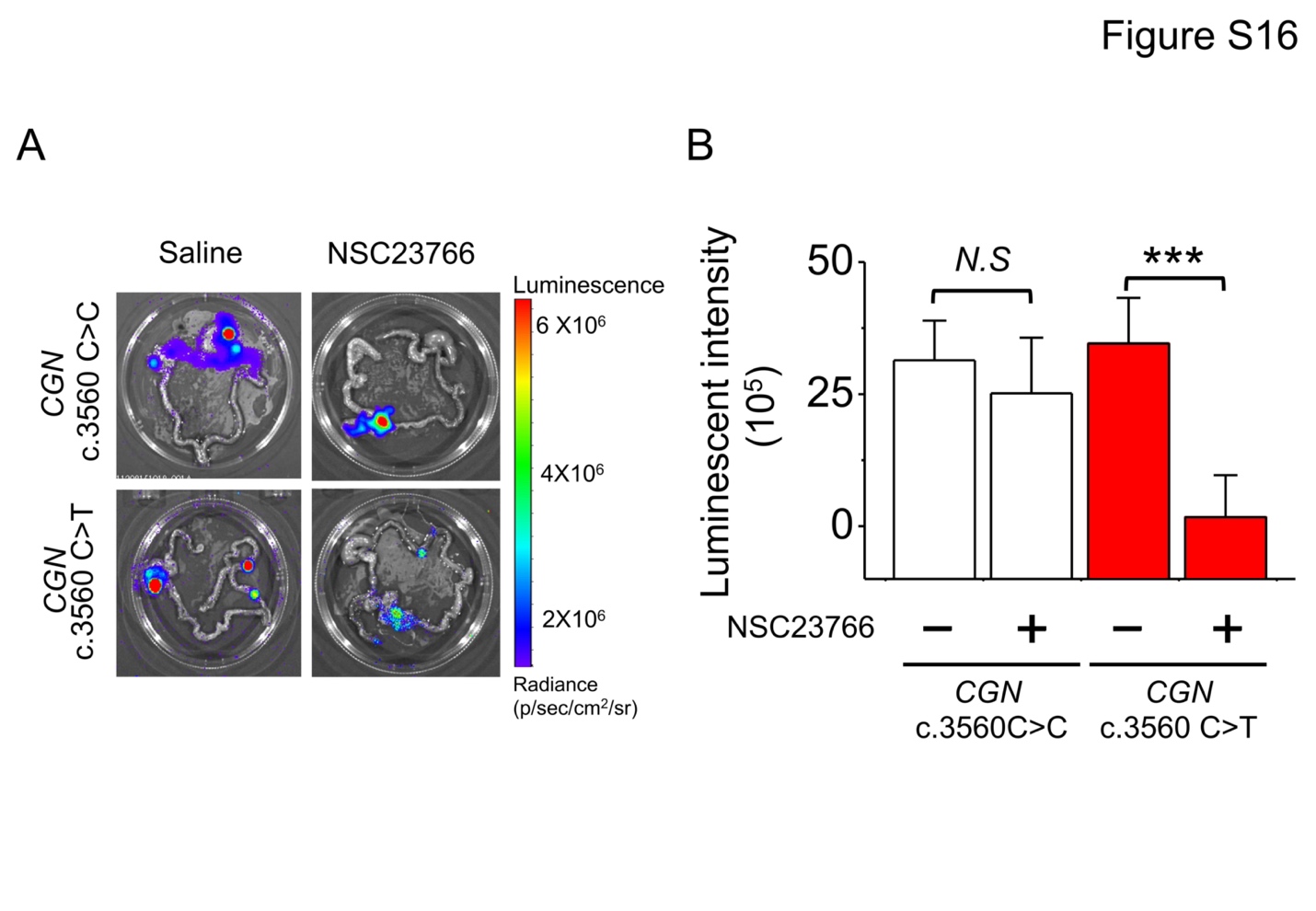


**Supplementary Fig. S16. The activity of intestinal tumors evaluated by IVIS in HT-29 orthotopic mouse model. A,** Representative images of the intensity of luminescent signals from the intestinal tumors in each group. The image was recorded on the 28th day post orthotopic xenograft. **B,** Quantitative analysis of signals from the IVIS luciferase images. Column, mean + SEM, n=6 in each group. *t*-test for statistical significance, ****p* < 0.001; N.S, ≥ 0.05, not significant.

**
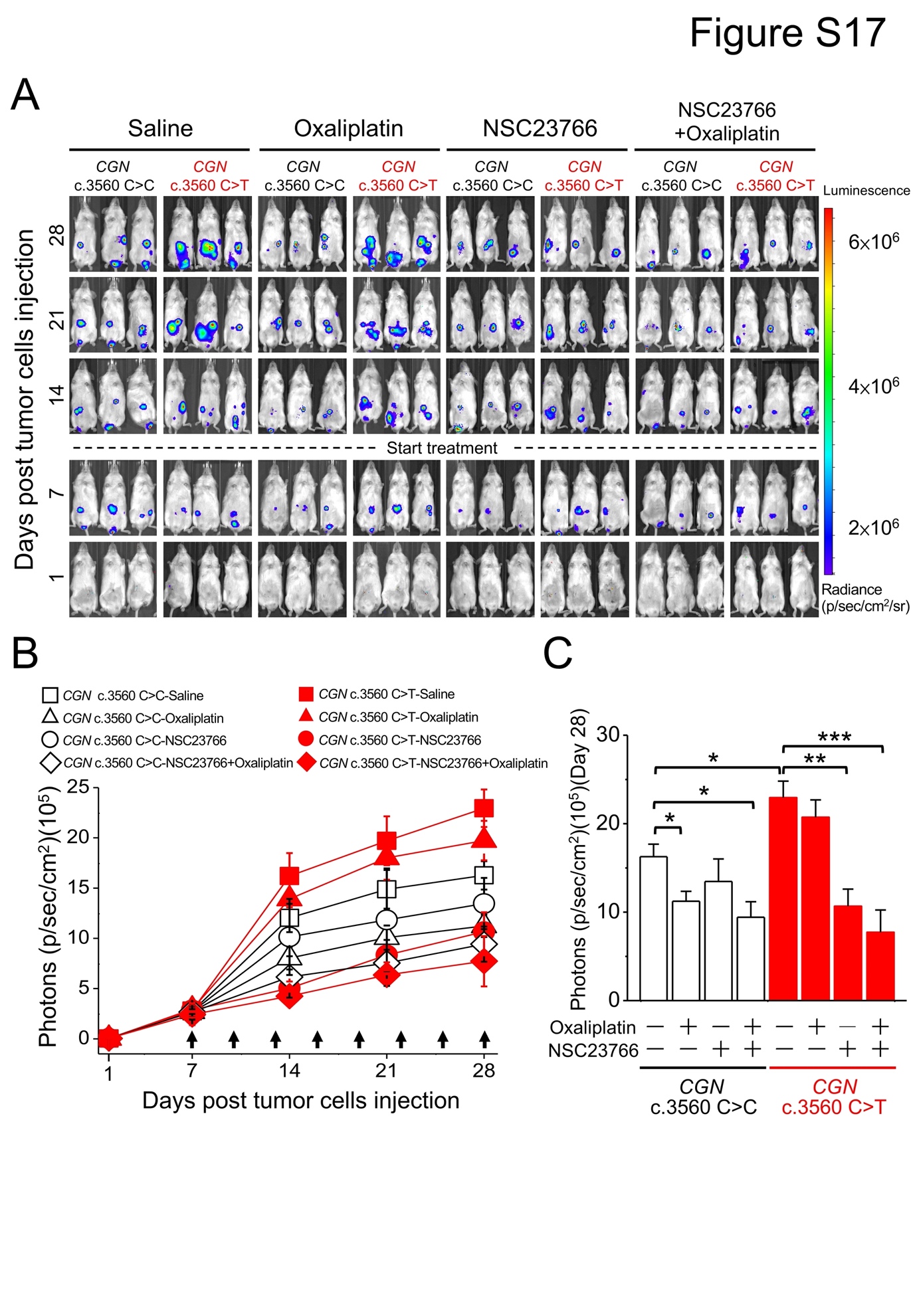
**

**Supplementary Fig 17. The efficacy of NSC23766 in tumor inhibition is influenced by the mutational status of *CGN*. A,** The progression of tumors in *CGN* WT and c.3560C>T HT-29 orthotopic xenografts were evaluated using the IVIS system, with representative images illustrating the effects of different treatments, including saline, NSC23766, oxaliplatin, or combination therapy. Luminescence signals were monitored through repeated imaging until the fourth week post-inoculation, and the data were presented as radiance (photons/sec/cm²/steradian) with a corresponding color bar. **B,** The luminescence intensity from each tumor was quantitatively analyzed using optical bioluminescence imaging at 1, 7, 14, 21, and 28 days post-injection of cancer cells. Arrows indicate the treatment time points. Value, mean + SEM, n=6 in each group. **C,** Quantitative analysis of signals from the IVIS luciferase images. Column, mean + SEM, n=6 in each group. *t*-test for statistical significance, **p* < 0.05; ***p* < 0.01; ****p* < 0.001.


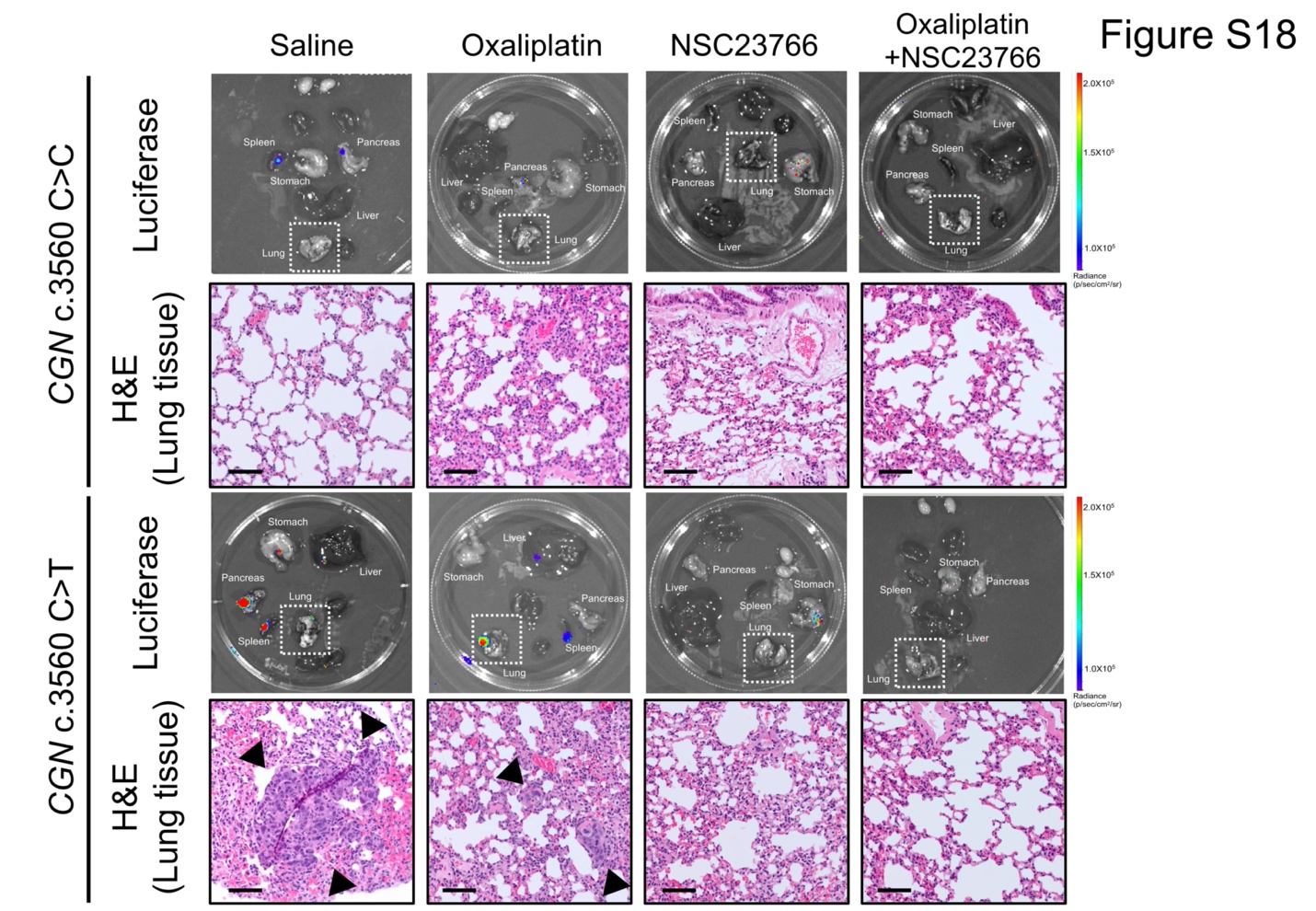


**Supplementary Fig 18. The metastasis status of *CGN* WT and c.3560 C>T orthotopic tumors in response to oxaliplatin, NSC23766, or their combination.** (Upper) Representative luciferase images of lungs, liver, stomach, spleen, and pancreas at the 28^th^ day post-orthotopic xenograft. (Lower) H&E staining of lung tissues in *CGN* c.3560 C>C group and *CGN* c.3560 C>T group. Lung metastatic lesions (black arrows) were observed in *CGN* mutant without NSC23766 treatment group. Photographs using the 20× objective. Scale bar, 100 μm.

**Supplementary Tables (separate file)**

**Supplementary Table S1.** Oncomine Comprehensive Assay v3 data of four tumor tissues from the proband.

**Supplementary Table S2.** Germline *BRCA1/2* profiling of the three affected members from the proband family.

**Supplementary Table S3.** The data of germline genome sequencing of three affected and two unaffected members from the proband’s family.

**Supplementary Table S4.** The characteristics of the 101 candidate variants.

**Supplementary Table S5.** The clinical information of 222 patients in the validation cohort.

**Supplementary Table S6.** Peptide sequences identified in a single spot on a 2D gel.

**Supplementary Table S7.** The antibodies used in the present study. WB, western blotting; Confocal, FV-3000 Confocal Laser Scanning Microscope. The dilutions used for each application are shown.
